# Genomic insights into deleterious mutations and their impact on agronomic traits during pear domestication

**DOI:** 10.1093/hr/uhaf140

**Published:** 2025-05-29

**Authors:** Xiang Zhang, Bobo Song, Shuai Du, Shiqiang Zhang, Yuexing Ren, Cheng Xue, Shaozhuo Xu, Pengfei Zheng, Shulin Chen, Zhiwen Qiao, Jiahao Liu, Wei Wei, Jun Wu

**Affiliations:** College of Horticulture Science and Engineering, Shandong Agricultural University, No. 61 Daizong Street, Tai'an, Shandong 271018, China; College of Horticulture, State Key Laboratory of Crop Genetics & Germplasm Enhancement and Utilization, Nanjing Agricultural University, No. 1 Weigang Street, Nanjing, Jiangsu 210095, China; Zhongshan Biological Breeding Laboratory, No. 50 Zhongling Street, Nanjing, Jiangsu 210014, China; College of Horticulture Science and Engineering, Shandong Agricultural University, No. 61 Daizong Street, Tai'an, Shandong 271018, China; College of Horticulture, State Key Laboratory of Crop Genetics & Germplasm Enhancement and Utilization, Nanjing Agricultural University, No. 1 Weigang Street, Nanjing, Jiangsu 210095, China; College of Horticulture Science and Engineering, Shandong Agricultural University, No. 61 Daizong Street, Tai'an, Shandong 271018, China; College of Horticulture Science and Engineering, Shandong Agricultural University, No. 61 Daizong Street, Tai'an, Shandong 271018, China; College of Horticulture Science and Engineering, Shandong Agricultural University, No. 61 Daizong Street, Tai'an, Shandong 271018, China; College of Horticulture Science and Engineering, Shandong Agricultural University, No. 61 Daizong Street, Tai'an, Shandong 271018, China; College of Horticulture Science and Engineering, Shandong Agricultural University, No. 61 Daizong Street, Tai'an, Shandong 271018, China; College of Horticulture Science and Engineering, Shandong Agricultural University, No. 61 Daizong Street, Tai'an, Shandong 271018, China; College of Horticulture Science and Engineering, Shandong Agricultural University, No. 61 Daizong Street, Tai'an, Shandong 271018, China; College of Horticulture Science and Engineering, Shandong Agricultural University, No. 61 Daizong Street, Tai'an, Shandong 271018, China; College of Horticulture, State Key Laboratory of Crop Genetics & Germplasm Enhancement and Utilization, Nanjing Agricultural University, No. 1 Weigang Street, Nanjing, Jiangsu 210095, China; Zhongshan Biological Breeding Laboratory, No. 50 Zhongling Street, Nanjing, Jiangsu 210014, China

## Abstract

The pear (*Pyrus* spp.), a perennial fruit tree, is subjected to genetic alterations over decades or even centuries to adapt to complex climatic and cultivation conditions. Genome-wide studies of deleterious mutations remain limited in perennial fruit trees, particularly regarding the effects of domestication on deleterious mutations. In this study, 232 pear accessions were resequenced, and 9 909 773 single-nucleotide polymorphisms (SNPs), and 139 335 deleterious mutation sites, were identified genome wide. A higher proportion of deleterious mutations in coding regions (1.4%) were observed in the pear genome than annual crops. During domestication, a reduction in deleterious mutations in *Pyrus pyrifolia*/*P. bretschneideri* was found to be associated with their decreases in selective sweep regions. Conversely, an increase in the number of deleterious mutations in *P. communis* was observed, which may be related to a higher occurrence within selective sweep regions. In *P. ussuriensis*, an overall increasing trend in deleterious mutations was identified, which was determined to be unrelated to domestication or gene introgression but instead linked to its relatively high heterozygosity. Differential deleterious mutation genes were identified during the domestication process. Among these, the *PyMYC2* gene, associated with stone cell synthesis, was identified through GWAS, overexpression of *PyMYC2* in pear callus significantly promoter lignin biosynthesis, *PyMYC2* contains three nonsynonymous deleterious mutations that were selected during the domestication of Asian pears. This research provides new insights into developing future breeding strategies aimed at improving agronomic traits and offers a framework for studying deleterious mutation patterns in the domestication of perennial fruit trees.

## Introduction

Mutations are recognized as the cornerstone of evolution [1]. Gene mutations introduce new genetic variations, which serve as the raw materials for natural selection and genetic drift. Evolution is driven by the selection and accumulation of these variations, enabling organisms to adapt and survive in constantly changing environments. New genetic variations are continually introduced into populations through mutations [2]. These variations exert a wide range of impacts on an individual’s fitness. Although a small fraction of mutations are beneficial [3], most are neutral or even lethal [4]. Typically, deleterious mutations alter highly conserved regions or lead to loss of protein function. Highly beneficial mutations are increased in frequency through positive selection until fixation occurs, while highly deleterious mutations are rapidly eliminated by purifying selection and are removed from the population. Mildly deleterious mutations, however, may persist within populations for extended periods, accumulating within genetic diversity and potentially reducing species fitness [2, 5].

In recent years, the identification of deleterious plant mutations has been increasingly studied. For instance, it has been reported that 20% of nonsynonymous mutations in rice [6] and *Arabidopsis* [7] are deleterious. Similarly, deleterious SNPs have been annotated in 19 and 8% of exon regions in maize and sorghum, respectively, accounting for 0.21 and 0.67% of the total SNPs [8]. In soybeans, a total of 315 029 deleterious nonsynonymous mutations were identified within the CDS region, comprising 1.0% of the total SNPs. It was observed that deleterious mutations decreased by 7.1% during domestication from wild to landrace varieties and further decreased by 1.4% during the improvement from landrace to cultivated varieties. The number of deleterious mutations was also found to have decreased in domestication sweep regions [9]. Notably, in tetraploid potatoes, clonal propagation was reported to result in the accumulation of numerous deleterious mutations in their genomes, which were partially or entirely masked in their heterozygous state [10, 11]. In seed-propagated diploid potatoes, 367 499 deleterious mutations were identified using evolutionary rate constraint methods, and the inclusion of deleterious mutation burden in models improved yield prediction accuracy by 24.7% [12]. The patterns of genome-wide deleterious mutations during the domestication and improvement of plants, especially perennial crops, are crucial for breeding programs but remain poorly understood.

The pear (*Pyrus* spp.), an important temperate economic fruit tree, originated in southwest China, spread throughout Central Asia, and eventually reached West Asia and Europe [13–15]. Pears are valued for their long cultivation history, rich nutritional content, and global consumer preference. Over 5000 pear germplasms have been recorded worldwide. The main cultivated pear varieties belong to the following four Asian pear species, including *Pyrus pyrifolia*, *P. bretschneideri*, *P. ussuriensis*, and *P. sinkiangensis*, as well as the European pear species *P. communis*, wherein each species exhibits diverse morphologies and ecological adaptability [15–18]. The publication of the first genome sequence of the Asian pear in 2013 marked a significant milestone, as it enhanced our understanding of chromosome evolution, genome structure and genetic variation patterns [19]. This advancement enabled the identification of numerous genes controlling important pear traits. Subsequent developments in stable and reliable molecular markers, the construction of new gene maps, the development of a 200 K SNP genotyping chip, and in-depth genome evolution analyses have revolutionized genetic improvement efforts for pears [20, 21]. With the maturation and cost reduction of high-throughput sequencing technologies, new sequencing and resequencing projects and in-depth transcriptome analyses have been conducted, providing extensive genomic information. These advances have significantly enhanced the understanding of plant domestication processes, revealing the genetic mechanisms underlying complex traits and opening new opportunities for agricultural technology innovation. However, the patterns of deleterious mutations during the prolonged domestication and improvement of pears remain unclear, necessitating further exploration.

In this study, a comprehensive analysis of deleterious mutations was conducted across 232 pear germplasm, including major cultivated and wild varieties. The aim was to investigate the patterns of deleterious mutations during the domestication of Asian and European pears. Our main findings include: (i) the genome-wide landscape of deleterious mutations within the pear genome, (ii) the differing patterns of deleterious mutation changes during the domestication of three different pear groups (*P. pyrifolia*, *P. ussuriensis*, and *P. communis*), and the underlying reasons for these differences, and (iii) the influence of deleterious mutation sites on genes and their effects on phenotypic traits. These findings provide new insights into the genetic mechanisms underlying pear domestication and offer valuable implications for future breeding strategies.

## Results

### Genome-wide variation, population structure, and genetic diversity

By combining published data and 84 new pear germplasm, resequencing data were obtained for 232 pear samples (Table S1). These samples included 15 varieties of *P. bretschneideri* (all cultivated), 62 of *P. pyrifolia* (15 wild and 47 cultivated), 32 of *P. ussuriensis* (22 wild and 10 cultivated), 5 of *P. sinkiangensis* (all cultivated), and 66 of *P. communis* (11 wild and 55 cultivated). Additionally, 28 Asian wild and 24 wild European germplasm were analyzed. A total of 889.6 GB of high-quality sequencing data was generated, with an average of 3.83 GB per sample and a sequencing depth of 10.7×. The sequences were aligned to the ‘Cuiguan’ genome [22], achieving an average alignment rate of 85.14% (Table S2). Across all samples, 9 909 773 SNPs were identified, with approximately 16.12% located in intron regions and 55.90% in intergenic regions. Among these variations, 5.57% were synonymous mutations, and 5.22% were nonsynonymous mutations (Table S2, Fig. S1, Fig. S2).

**Figure 1:**
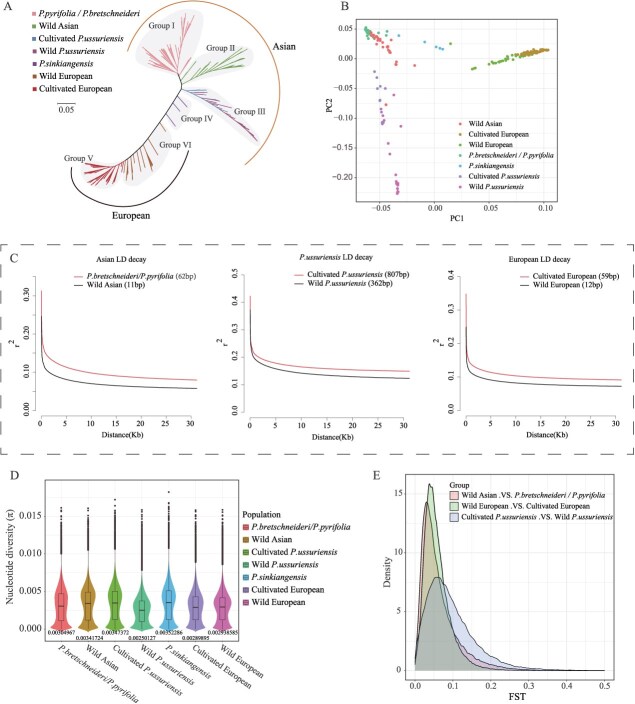
Genetic diversity analysis of different pear populations. A. Phylogenetic tree of 232 pear germplasm. B. PCA plot of the first two principal components for 232 pear germplasm. C. Linkage disequilibrium (LD) decay analysis for three comparisons: Comparison A (*P. pyrifolia*/*P. bretschneideri* vs. wild Asian), Comparison B (cultivated *P. ussuriensis* vs. wild *P. ussuriensis*), and Comparison C (cultivated European vs. wild European). The *x*-axis represents physical distance (kb), and the *y*-axis represents the LD coefficient (*r*^2^). D. Nucleotide diversity (π) statistics for seven pear populations. The *y*-axis represents π, while the *x*-axis shows populations in the following order: *P. pyrifolia*/*P. bretschneideri*, wild Asian, cultivated *P. ussuriensis*, wild *P. ussuriensis*, *P. sinkiangensis*, cultivated European, and wild European. E. Distribution density of *F*st values for the same three comparisons as in panel C. The *x*-axis represents *F*st values, and the *y*-axis shows distribution density.

Phylogenetic analysis of the SNPs revealed two primary categories of pear germplasm: Asian and European pears. Principal component analysis further confirmed a clear distinction between the two groups (Fig. 1A). Asian pears were divided into four groups: *P. pyrifolia* and *P. bretschneideri* (Group I), which clustered closely due to the domestication of *P. bretschneideri* and *P. pyrifolia*. Wild germplasm from various regions of Asia (Group II), including a subset of wild *P. pyrifolia*. Shared backgrounds among *P. pyrifolia*, *P. bretschneideri*, and wild Asian germplasm were evident from the population structure analysis (Figs 1B and Fig. S3B). *P. ussuriensis* (Group III), comprising both cultivated and wild types from cold regions. Some cultivated and wild types are intermixed phylogenetically, while others are more distantly related (Fig. 1A and B), suggesting that *P. ussuriensis* should be analyzed as an independent population. *P. sinkiangensis* (Group IV), a hybrid of Asian and European pears, was likely introduced to Xinjiang during the Han Dynasty through the Silk Road [23, 24]. This group exhibits a mixed genetic background, with components from both Asian and European pears (Fig. S3B).

European pears were categorized into *P. communis* (Group V) and wild European pears (Group VI), which displayed relatively homogeneous genetic backgrounds (Fig. S3A and B).

### Linkage disequilibrium and genetic diversity

Linkage disequilibrium (LD) decay rates were assessed across pear populations. Wild populations exhibited faster LD decay than cultivated populations, with wild Asian pears showing the fastest decay (11 bp) while cultivated *P. ussuriensis* the slowest (807 bp). Among cultivated populations, *P. ussuriensis* had significantly greater LD decay distance than other groups (Fig. 1C). Pears, in general, showed much faster LD decay rates than other annual crops and fruit trees, such as *Oryza sativa indica* (123 kb), *O. sativa japonica* (167 kb) [25, 26], maize (10–50 kb) [27], foxtail millet (100 kb) [28], *Vitis sylvestris* (western) (1.0–1.6 kb) [29]. This rapid decay reflects the short domestication history of pears, fewer selection pressures, and high genetic recombination due to their self-incompatible, cross-pollinating nature.

Nucleotide diversity (π) was highest in *P. sinkiangensis*, consistent with its hybrid origin, which promotes genetic recombination. The diversity of *P. pyrifolia*/*P. bretschneideri* (0.00305) was slightly lower than in wild Asian pears (0.00341), and cultivated European pears (0.00290) showed marginally lower diversity than wild European pears (0.00293) (Fig. 1D). Domestication typically reduces nucleotide diversity; however, cultivated *P. ussuriensis* exhibited increased diversity, contrary to expectations. This suggests unique domestication patterns and highlights that *P. pyrifolia*/*P. bretschneideri,* wild Asian pears, and *P. ussuriensis* populations should not be analyzed as a single group.

The fixation index *F*st indicated the least differentiation between *P. pyrifolia*/*P. bretschneideri* and wild Asian pears, with the highest differentiation observed between cultivated and wild *P. ussuriensis* (Fig. 1E). Overall, the low *F*st values across populations reflect a low level of domestication in pears compared to other species such as millet (0.02–0.03) [30] and *Juglans regia* (0.11–0.21) [31]. In *P. ussuriensis*, the higher nucleotide diversity in cultivated populations may result from human selection introducing new genetic variations, frequent gene flow, and larger population sizes (Fig. S9A).

### Selective signaling and convergent domestication in different pear populations

To identify regions subjected to human selection during domestication, three comparisons were performed using wild populations as the background: Comparison A (*P. pyrifolia*/*P. bretschneideri* vs. wild Asian), Comparison B (cultivated *P. ussuriensis* vs. wild *P. ussuriensis*), and Comparison C (cultivated European vs. wild European). A cross-population composite likelihood ratio (XP-CLR) test detected genomic regions with extreme allele frequency differentiation in cultivated populations. The top 5% of intervals identified by all scanning methods were considered domestication-selected regions, and genes within these regions were subjected to GO and KEGG enrichment analyses.

**Figure 2:**
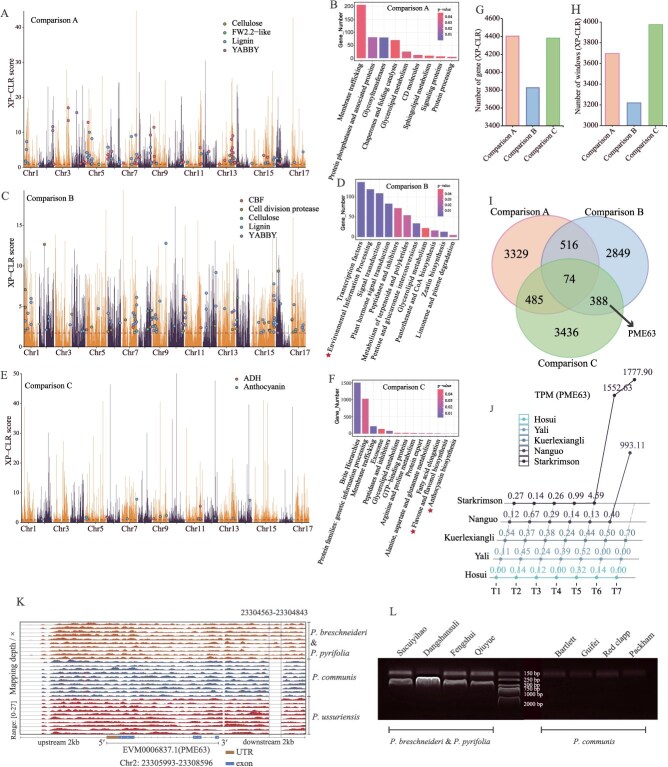
Selection and domestication signals in different pear populations. A. Genome-wide XP-CLR values for Comparison A (*P. pyrifolia*/*P. bretschneideri* vs. wild Asian); regions above the dashed line (XP-CLR > 0.8939272) are identified as selection and domestication regions. B. KEGG enrichment analysis of genes in the selection and domestication regions for Comparison A. The *x*-axis shows KEGG terms, the *y*-axis shows the number of genes, and the color indicates *P*-values. C. Genome-wide XP-CLR values for Comparison B (cultivated *P. ussuriensis* vs. wild *P. ussuriensis*); regions above the dashed line (XP-CLR > 1.7214569) are identified as selection and domestication regions. D. KEGG enrichment analysis of genes in the selection and domestication regions for Comparison B. E. Genome-wide XP-CLR values for Comparison C (cultivated European vs. wild European); regions above the dashed line (XP-CLR > 0.9186784) are identified as selection and domestication regions. F. KEGG enrichment analysis of genes in the selection and domestication regions for Comparison C. G. Number of selected genes in each comparison group. H. Number of selection scan signals merged across all three groups. I. Venn Diagram showing overlap of selected genes between the three groups; *PME63* is identified in both Comparisons B and C. J. Expression Levels of *PME63* across seven developmental stages for five pear varieties (TPM); T1: fruit setting, T2: physiological fruit dropping, T3: rapid fruit enlargement, T4: one month after fruit enlargement, T5: prematurity, T6: maturity, T7: postharvest fruit senescence. K. Sequencing depth of *PME63* upstream, gene body, and downstream regions. Yellow represents *P. pyrifolia*/*P. bretschneideri*, blue represents *P. communis* and red represents *P. ussuriensis*. L. The DNA cloning verification of 4 *P. pyrifolia*/*P. bretschneideri* and 4 *P. communis* to check if SV exists.

In Comparison A, enrichment was observed in biological processes such as multicellular organism development, postembryonic development, flower development, reproduction, and cell growth (Figs 2B and S4A). Genes influencing fruit size, sugar, and acid content-including starch synthase, YABBY, *fw2.2* [32], and *PbrNSC* (EVM0015970.1) were identified [33], alongside lignin and cellulose synthesis-related genes (Fig. 2A). Comparison B revealed enrichment in processes such as postembryonic development, multicellular organism development, flower development, plant hormone signal transduction, environmental information processing, and responses to abiotic and light stimuli (Figs 2D and S4A). This indicates adaptations to high-altitude or cold regions. Key genes included members of the CBF family associated with stress resistance [34–36] and genes related to sugar and lignin synthesis (Fig. 2C). Comparison C highlighted anthocyanin, flavonoid, and flavonol biosynthesis pathways (Figs 2F and S4A), suggesting an emphasis on improving color and flavor in European pears. Genes related to aroma biosynthesis and sugar-acid content were identified (Fig. 2E). Interestingly, sugar- and acid-related genes were more prevalent in European pears, while lignin- and cellulose-related genes were more abundant in Asian pears and *P. ussuriensis*, aligning with their higher stone cell content and distinct textures and flavors.

Population- and chromosomal-level selective signals revealed varying levels of selection on different chromosomes (Fig. S5). The highest number of selective signals was observed in Comparison C (3975), while the lowest was in Comparison B (3219) (Fig. 2H). The number of selected genes in Comparison A (4404) was similar to Comparison C (4383), whereas Comparison B (3827) had the fewest selected genes (Fig. 2G). Only 74 genes were commonly selected across all three populations, with limited overlap between any two populations (Fig. 2I), suggesting unbalanced selection trends.

A convergently domesticated gene, *PME63* (EVM0006837.1), was identified in Comparisons B and C (Fig. 2I). *PME63* has been linked to pear fruit softening [37] and exhibited high expression exclusively during the late developmental stages of *P. ussuriensis* and *P. communis* (Fig. 2J). Structural variation (SV) of approximately 300 bp was detected within 2 kb downstream of *PME63*, specifically in wild *P. ussuriensis* and *P. communis* (Fig. 2K). In *Brassica oleracea*, a 316 bp SV downstream of the *BoCKX3* gene leads to a significant increase in the expression level of this gene [38]. PCR amplification confirmed the presence of this SV in *P. communis* but not in *P. breschneideri* or *P. pyrifolia* (Fig. 2L, Table S3). This SV may drive the specific late-stage expression of *PME63* in *P. ussuriensis* and *P. communis*, contributing to their ripening and softening processes and explaining differences in fruit softening between Eastern and Western pear varieties.

### Patterns of deleterious mutations across the pear genome

**Figure 3:**
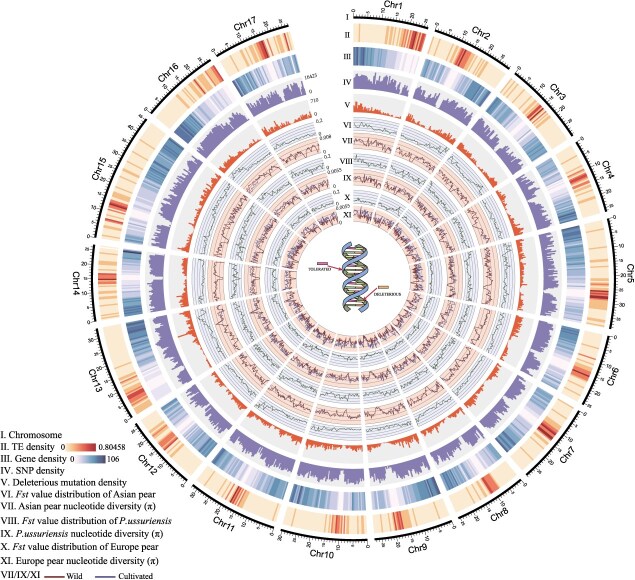
Circos plot of pear genome-wide characteristics. I. Chromosomes. II. Transposable element density. III. Gene density. IV. SNP density. V. Deleterious mutation density. VI. Genome-wide *F*st values of Comparison A. VII. Nucleotide diversity (π) of Comparison A. VII. Genome-wide *F*st values of Comparison B. IX. π of Comparison B. X. Genome-wide *F*st values of Comparison C. XI. π of Comparison C.

In this study, patterns of deleterious mutations were assessed across the pear genome SIFT4G to evaluate the conservation of sequences (Fig. 3A–K). The distribution of deleterious mutations was found to be nonuniform, with mutations concentrated in the chromosome arm rather than the centromeric areas. High gene density did not necessarily correlate with increased densities of deleterious mutations, suggesting that their accumulation occurred within specific genes, and it was not evenly distributed.

**Figure 4:**
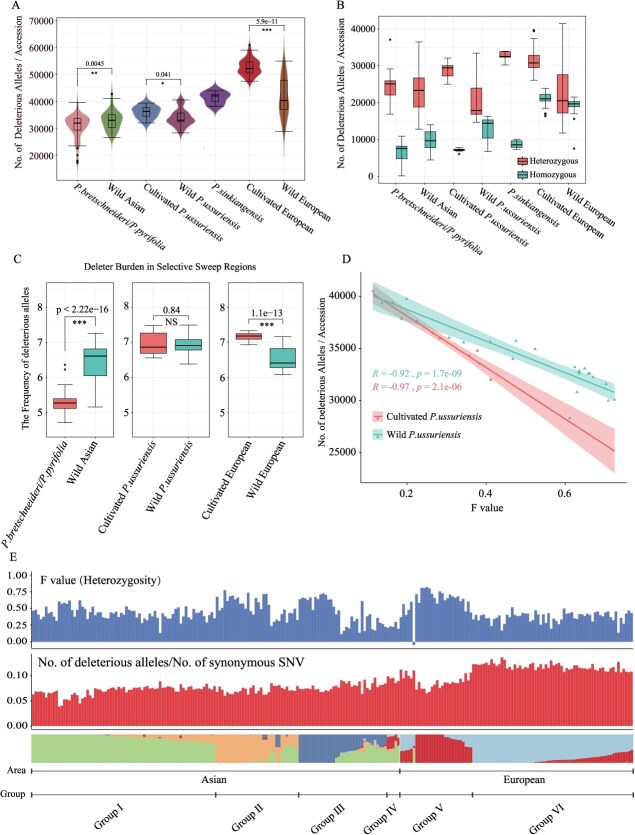
Patterns of deleterious mutations in different pear populations. A. Total number of deleterious mutations across seven pear populations. The *y*-axis shows the total number, and the *x*-axis populations in the order, from left to right: *P. pyrifolia*/*P. bretschneideri*, wild Asian, cultivated *P. ussuriensis*, wild *P. ussuriensis*, *P. sinkiangensis*, cultivated European, and wild European. Significant differences are shown for the three comparisons, with *P*-values indicated. B. Number of homozygous and heterozygous deleterious mutations in each population. C. Percentage of deleterious mutations in selection/domestication regions for each comparison. The *y*-axis represents the percentage of deleterious mutations in the selection domestication interval as a proportion of the total deleterious mutations, with Comparison A, Comparison B, and Comparison C groups shown from left to right and *P*-values displayed above the bars. D. Correlation between deleterious mutation number and heterozygosity (F) for Comparison B. E. Top: Heterozygosity (F) for each individual. Middle: Ratio of deleterious mutations to synonymous mutations. Bottom: Population structure at K = 5, with samples ordered based on structure. ^*^P < 0.05, ^**^P < 0.01, ^***^P < 0.001, ^****^P < 0.0001; ns, not significant (Student’s t-test).

Cultivated European varieties exhibited the highest number of deleterious mutations, while *P. pyrifolia*/*P. bretschneideri* had the lowest, and *P. sinkiangensis* displayed intermediate levels (Fig. 4A and E). Validation using GERP, an evolutionary constraint-based method, yielded consistent trends with *F*st, though fewer deleterious mutations were identified by GERP (Fig. S6A). Approximately one-third of GERP-identified sites overlapped with those detected by SIFT4G (Fig. S6B), with SIFT4G results primarily used for subsequent analyses. A significant reduction in deleterious mutations was observed in *P. pyrifolia*/*P. bretschneideri* compared to wild Asian varieties (*P* = 0.0045), while cultivated varieties in other comparisons showed increased deleterious mutations compared to their corresponding wild types (*P* = 0.041; *P* = 5.9e-11) (Fig. 4A).

Deleterious mutations were predominantly heterozygous across populations, with cultivated varieties exhibiting more heterozygous and fewer homozygous mutations compared to wild types, with the exception of wild European pear. This suggests that domestication may have introduced more heterozygous deleterious mutations (Fig. 4B), similar to patterns observed in potatoes [10].

The frequency of deleterious mutations in selection intervals was consistent with whole genome patterns (Fig. S7). In *P. pyrifolia*/*P. bretschneideri*, human selection appeared to reduce deleterious alleles (*P* < 2.22e-16) (Fig. 4C), resembling trends observed in soybeans. No significant differences were detected in *P. ussuriensis* (*P* = 0.84) (Fig. 4C), while cultivated European varieties exhibited an increase in deleterious mutations due to domestication (*P* = 1.1e-13) (Fig. 4C).

Heterozygosity levels (*F* values) were analyzed to explore the patterns of deleterious mutation changes. Wild populations displayed higher *F* values, indicating lower heterozygosity compared to cultivated varieties (Figs S8A and 4E). A significant negative correlation was identified between the number of deleterious mutations and *F* values, particularly in cultivated *P. ussuriensis* (*R* = −0.97, *P* = 2.1e-06) (Fig. 4D).

The role of introgression in *P. ussuriensis* was also examined using D-statistics and fd-statistics. Significant gene flow was detected between *P. pyrifolia*/*P. bretschneideri* and cultivated *P. ussuriensis*) (*D* = 0.3364, D *Z* score = 57.723) (Fig. S9A). The fd-statistics indicated significant introgression across all 17 chromosomes of pears (Fig. S9B). However, no significant differences in deleterious mutation frequency were observed in introgression intervals (*P* = 0.38) (Fig. S9D), suggesting that introgression does not affect deleterious mutation patterns in cultivated *P. ussuriensis*.

### Identification and functional analysis of differentially deleterious mutated genes

To determine the biological significance of deleterious mutations, three populations were analyzed by comparing the number of deleterious mutation sites in all genes of cultivated varieties with their respective wild counterparts. A method similar to identifying differentially expressed genes in transcriptome studies was employed to identify differentially deleterious mutated genes (DDMGs). Genes with |log_2_(FoldChange)| ≥ 1 and *P*_adj_ ≤ 0.05 were classified as DDMGs.

**Figure 5:**
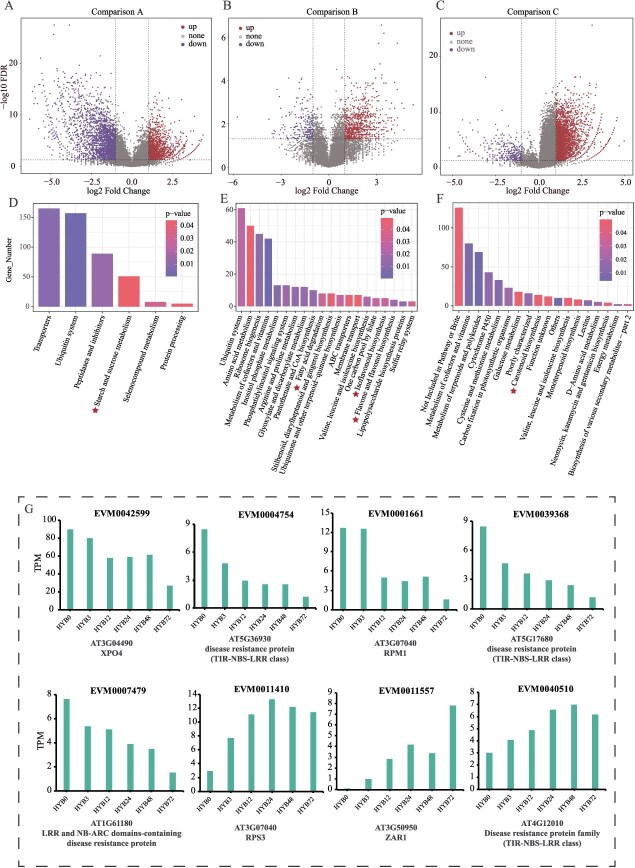
Differentially deleterious mutated genes (DDMGs) in pear populations. A-C. Volcano plots of DDMGs for Comparisons A, B, and C. D-F. KEGG pathway enrichment analysis of DDMGs for Comparisons A, B, and C; the *x*-axis shows KEGG terms, the *y*-axis shows gene count. G. Expression changes of eight randomly selected NB-ARC family genes at different time points after inoculation with fire blight bacteria, with gene ID and *Arabidopsis* genes shown*.*

In Comparison A (*P. pyrifolia*/*P. bretschneideri* vs. wild Asian), 4898 DDMGs were identified, including 3130 downregulated and 1768 upregulated genes (Fig. 5A). In Comparison B (cultivated *P. ussuriensis* vs. wild *P. ussuriensis*), 1900 DDMGs were detected, comprising 423 downregulated and 1477 upregulated genes (Fig. 5B). In Comparison C (cultivated European vs. wild European), 4635 DDMGs were identified, including 392 downregulated and 4243 upregulated genes (Fig. 5C). These DDMGs were further annotated to assess their potential biological significance.

KEGG Pathway enrichment analysis was performed for the DDMGs across the three groups. In Comparison A, the enriched pathways included starch and sucrose metabolism (Fig. 5D), suggesting that deleterious mutations likely affected fruit size, sugar metabolism, and related processes during domestication. In Comparison B, enriched pathways included fatty acid degradation, flavone and flavonol biosynthesis, and isoflavonoid biosynthesis (Fig. 5E), indicating potential effects on fruit softening, coloration, and stress resilience. For Comparison C, carotenoid biosynthesis was the most enriched pathway (Fig. 5F), suggesting a greater impact of deleterious mutations on fruit color during domestication.

Since SIFT 4G evaluates the functional impact of mutations based on sequence homology and species conservation, it predicts whether specific amino acid substitutions are detrimental by comparing pre- and postmutation sequences [39]. However, protein function and stability are influenced by multiple factors, including three-dimensional structure, protein–protein interactions, and the cellular environment. It is challenging to accurately predict the effects of a mutation at a single site alone. Thus, even if a mutation is predicted to be deleterious, its actual impact may be context-dependent and, in some cases, beneficial.

For example, among the upregulated DDMGs in Comparison A, 26 structural genes related to lignin biosynthesis and 3 related to cellulose biosynthesis were identified. Conversely, among the downregulated DDMGs, 25 lignin biosynthesis-related genes and 4 cellulose biosynthesis-related genes were detected (Table S4). In Comparison B, upregulated DDMGs included 21 lignin biosynthesis-related genes and 1 cellulose biosynthesis-related gene, while the downregulated DDMGs included 6 lignin biosynthesis-related genes and 1 cellulose biosynthesis-related gene (Table S5). These findings suggest that the reduced stone cell content in cultivated pears compared to wild pears may be linked to deleterious mutations impairing the function of these genes (Fig. S10A).

Moreover, among the upregulated DDMGs in Comparison C, 65 genes belonging to the NB-ARC family were identified (Table S6, Fig. S10B–D). These genes are widely reported to play roles in plant disease resistance, and the accumulation of deleterious mutations in these genes may contribute to the relatively poor disease resistance observed in European cultivated pears.

In summary, deleterious mutations typically involve changes at evolutionarily conserved sites, potentially impacting gene or protein function. However, in agricultural contexts, their impacts may vary and should be evaluated individually. Identifying deleterious mutation sites could provide valuable guidance for future pear breeding programs.

Additionally, eight NB-ARC family genes from the upregulated DDMGs in Comparison C were selected, and their expression dynamics were analyzed using transcriptome data from a published study on fire blight pathogen inoculation [40]. Data were collected at 3-, 12-, 24-, 48-, and 72-h postinoculation, along with a control (CK). Among these genes, five exhibited a gradual decrease in expression after pathogen inoculation, whereas three showed a gradual increase (Fig. 5G). The expression changes suggest that these genes play roles in pathogen response and disease resistance. Consequently, the accumulation or reduction of deleterious mutations in these genes could alter their functionality.

### The impact of deleterious mutations on agronomic traits

Stone cells, a distinguishing trait of pears compared to other Rosaceae plants, are primarily formed through secondary cell wall deposition on the primary cell wall and are closely associated with the thickening of parenchyma cell walls. The content of stone cells directly influences the texture of pear fruits and is closely associated with lignin and cellulose biosynthesis, transfer, and deposition [41, 42]. *PbrNSC* has been identified as a positive regulator of lignin and cellulose biosynthesis in pear fruits. This gene transcriptionally activates several target genes involved in the formation of the secondary cell wall, such as *PbrMYB169*, *Pbr4CL4*, *PbrMYB132*, *PbrLAC4*, *PbrLAC5*, *PbrCESA4b*, *PbrCESA7a*, and *PbrCESA8a* [33, 43].

In this study, 353 resequencing datasets and 188 transcriptome sequencing datasets of *P. pyrifolia* were analyzed. From these data, 6 887 313 and 844 462 high-quality SNPs (minor allele frequency ≥ 5%, missing rate ≤ 70%) were identified, respectively. The deleterious mutation sites among these SNPs were predicted using SIFT4G*,* which scored the deleteriousness of SNP sites based on their conservation.

**Figure 6:**
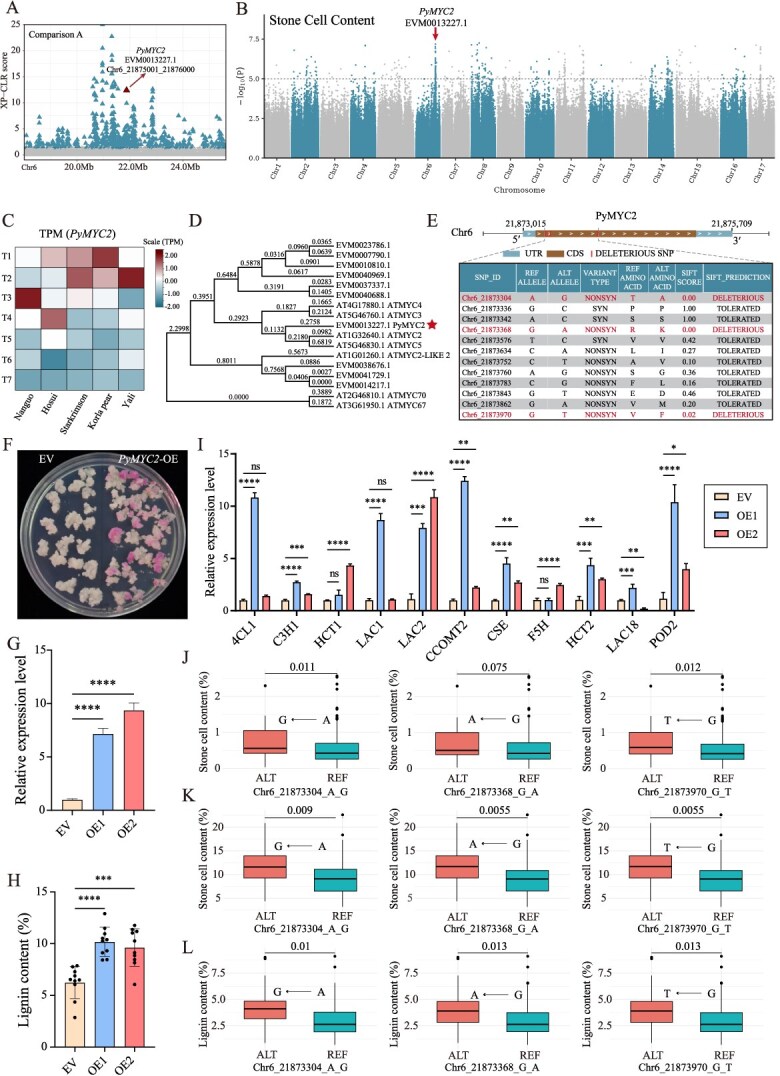
Deleterious mutation analysis of the gene *PyMYC2* (EVM0013227.1). A. Manhattan plot of XP-CLR scores for chromosome 6 from (18 000 000–25 626 161 bp); the *x*-axis shows the physical coordinates, the *y*-axis shows the XP-CLR score, and the dashed line indicates the selection threshold (0.8939272). B. The Manhattan plot of stone cells. The genome-wide significance threshold line (1e-5) is indicated by a dashed line, and a gene that is homologous to *AtMYC2*, *PyMYC2* (EVM0013227.1), is located in the GWAS peak region. C. Heatmap of *PyMYC2* expression levels across five pear qualities and seven developmental stages (TPM). T1: fruit setting, T2: physiological fruit dropping, T3: rapid fruit enlargement, T4: one month after fruit enlargement, T5: prematurity, T6: maturity, T7: postharvest fruit senescence. D. The phylogenetic relationship between *PyMYC2* and *Arabidopsis MYC2/3/4*. E. Top: Gene structure of *PyMYC2*. Bottom: Partial SNP information *PyMYC2* CDS. F. Phenotypes of transgenic pear calli stained with Wiesner’s reagent. G. Analysis of *PyMYC2* expression levels in transgenic pear calli transformed with EV or a *PyMYC2*-OE construct (lines OE-1 OE-2), mean ± SD (*n* = 3). H. Lignin contents were measured in transgenic pear calli with acetyl bromide, mean ± SD (*n* = 10). I. Relative expression of SCW biosynthesis genes in transgenic pear calli, mean ± SD (*n* = 3). J. Box plots of stone cell contents by genotypes (SNP1, SNP2, SNP3). ALT represents deleterious mutation genotype, REF represents nonmutation. Obtained using 353 resequenced *P. pyrifolia* genotypes and their phenotypic data. K-L. Box plots of stone cell and lignin contents by genotypes (SNP1, SNP2, SNP3). ALT represents deleterious mutation genotype, REF represents nonmutation. Obtained using 188 transcriptome sequencing genotypes of *P. pyrifolia* and their phenotypic data. ^*^P < 0.05, ^**^P < 0.01, ^***^P < 0.001, ^****^P < 0.0001; ns, not significant (one-way ANOVA, Dunnett post hoc test).

In this study, the gene *PyMYC2* (EVM0013227.1) was found within a selected domestication interval on chromosome 6 (Chr6_21865001_21875000) (Fig. 6A). This gene was also recognized as downregulated DDMG in Comparison A (Fig. 5A, Table S4). Based on the results of previous studies, we conducted a genome-wide association study (GWAS) using resequencing data from 353 pears and identified a significant peak associated with stone cell content on chromosome 6, with the estimated region being 20.18 MB–22.03 MB, which includes the gene *PyMYC2* (EVM0013227.1) (Fig. 6B). Its expression was primarily observed during the early stages of fruit development (Fig. 6C), consistent with the pattern of stone cell synthesis. Homology analysis indicated that *PyMYC2* is related to *Arabidopsis thaliana* genes *ATMYC2*, *ATMYC3*, and *ATMYC4* (Fig. 6D). Previous research has demonstrated that *ATMYC2/4* directly binds to the promoter of *ATNST1*, thereby activating its expression and promoting lignin and cellulose accumulation [44]. These findings suggest that *PyMYC2* may be a key gene regulating stone cell content in pear fruits.

To validate the aforementioned hypothesis, pear callus cultures were transformed with the *PyMYC2* overexpression (OE) construct or an empty vector (EV). Following induction with epibrassinolide (EBR) but without 2,4-D or 6-BA, phloroglucinol-HCl staining revealed strong red color in *PyMYC2*-OE, whereas the EV transformants showed only weak staining (Fig. 6F). Reverse transcription quantitative PCR (RT-qPCR) results demonstrated that *PyMYC2* was successfully overexpressed in two lines (OE1 and OE2) (Fig. 6G), with their lignin content significantly increased compared to the control group (Fig. 6H). Additionally, the expression levels of many lignin biosynthesis related genes (namely *4CL1*, *C3H1*, *HCT1*, *LAC1*, *LAC2*, *CCOMT2*, *CSE*, *F5H*, *HCT2*, *LAC18*, *POD2*) were notably upregulated in these lines (Fig. 6I). These results indicate that *PyMYC2* is a key regulatory gene controlling stone cells and lignin biosynthesis in pear fruit.

Three nonsynonymous SNPs within the CDS region of *PyMYC2* were identified as highly conserved (Fig. 6E): SNP1 (Chr6_21873304), SNP2 (Chr6_21873368), and SNP3 (Chr6_21873970). These SNPs were predicted to significantly alter gene function.

The relationship between these SNPs and phenotypic traits was further explored. We first statistically analyzed the genotype and phenotype data of 353 resequencing datasets of *P. pyrifolia* [45, 46], and found that the mutation of SNP1 from A to G (amino acid change: threonine to alanine, SIFT score = 0) significantly increased the content of stone cells (*P* = 0.011), while the mutation of SNP3 from G to T (amino acid change: valine to phenylalanine, SIFT score = 0.02) also led to a significant increase in stone cell content (*P* = 0.012). The change in SNP2 did not significantly alter the content of stone cells (*P* = 0.075) (Fig. 6J).

To verify the accuracy of our experimental conclusions, we also statistically analyzed the genotype and phenotype data from 188 previously published transcriptome sequencing datasets of *P. pyrifolia*. The mutation of SNP1 from A to G significantly increased the content of stone cells (*P* = 0.009) and lignin (*P* = 0.01). Similarly, the mutation of SNP2 from G to A (amino acid change: arginine to lysine, SIFT score = 0) and the mutation of SNP3 from G to T also led to significant increases in the content of stone cells (*P* = 0.0055) (Fig. 6K), lignin (*P* = 0.013) (Fig. 6L). These SNPs are considered potential causative mutations of *PyMYC2*.

The ALT genotype (deleterious mutation) of these SNPs was predominantly observed in wild populations, whereas the REF genotype (non-mutation) was mainly found in cultivated populations (Fig. S11). This distribution suggests that human selection during domestication likely reduced deleterious mutations in this gene, resulting in lower stone cell content in cultivated varieties.

In addition, a homologous gene, *PyNSC*-like (EVM0005971.1, score = 748, *P* = 0), was identified in 188 previously published transcriptome sequencing datasets of *P. pyrifolia*. Its expression levels were high during early fruit development in ‘Liyuanxiangli’ (a high stone cell variety) but lower in ‘Rongshan’ (a medium stone cell variety) and ‘Cuiguan’ (a low stone cell variety) (Fig. S12). This expression pattern aligns with stone cell biosynthesis, suggesting a functional similarity to *PbrNSC*.

A nonsynonymous SNP (Chr14_7813856) was identified within the CDS sequence of *PyNSC*-like. Mutation at this site from A to T (SIFT score = 0.02) was predicted to be deleterious (Fig. S13A–C). Samples with the T allele (deleterious mutation) exhibited significantly higher stone cell (*P* = 8.7e-05), lignin (*P* = 0.00013), and cellulose (*P* = 1.9e-08) content compared to samples with the A/A genotype (Fig. S13D). This SNP was proposed as a potential causative site for *PyNSC*-like.

## Discussion

### Pear exhibits higher burden of deleterious mutations

Research on deleterious mutations has been conducted across many plants, revealing the impact on introgression in wheat and grapes [47, 48], the identification and quantification of deleterious mutations in potatoes [12], and patterns influenced by domestication in soybeans, rice, and maize [8, 9, 49]. Our study provides a comprehensive analysis of deleterious mutations across the pear genome, offering new insights into their roles in domestication and impacts on agronomic traits. These findings highlight the intricate relationship between human and natural selection, genetic diversity, and the alteration in deleterious mutation, especially in perennial fruit crops such as pears. By analyzing genome-wide genetic variations across 232 pear germplasm, we identified distinctive genomic features in pear species. The proportion of nonsynonymous mutations in pears (5.22%) is higher than in some annual plants, such as soybean (1.9%) [50] and *Brassica oleracea* (4.0%) [51], closely matching previous studies [15]. However, it is lower than in some other annual plants, such as rice (10.1%) [52] and *B. rapa* (7.1%) [51]. Interestingly, we observed a higher proportion of deleterious mutations in pear compared to most annual plants. Specifically, deleterious SNPs accounted for 1.406% of total SNPs in pear, exceeding those reported in soybean (1.0%) [9], potato (0.6%) [12], maize (0.21%), and sorghum (0.67%) [8].

### The impact of domestication on deleterious mutations

In perennial fruit trees, traditional breeding methods face constraints due to their long growth cycles and high resource demands [53–56]. Molecular marker-assisted breeding has emerged as a more effective breeding strategy; yet, the linkage effects of genomic regions often introduce deleterious mutations alongside beneficial traits [57]. Identifying these deleterious mutations is thus critical. By categorizing pears into three main populations, each including both cultivated and wild varieties, we identified distinct deleterious mutation patterns during domestication and improvement. In *P. pyrifolia*/*P. bretschneideri*, cultivated varieties exhibited a reduction in deleterious mutations compared to their wild counterparts. This reduction aligns with findings in soybeans, where domesticated varieties showed a decrease in load compared to wild germplasm [9]. Such reductions are likely driven by decreased hitchhiking of deleterious alleles in linkage regions associated with selected traits. Conversely, in *P. communis*, cultivated varieties retained more deleterious mutations, similar to findings in rice and maize [8, 49], supporting the domestication cost hypothesis [58]. This hypothesis posits that domestication processes accumulate deleterious alleles through selection bottlenecks and genetic drift. *P. pyrifolia*/*P. bretschneideri* and *P. communis* exhibited contrasting patterns of deleterious mutations, which may be attributed to their distinct domestication targets, reflecting the pleiotropic effects of human selection. Interestingly, *P. ussuriensis* showed no significant correlation between deleterious mutations and domestication or gene introgression but exhibited a relationship with heterozygosity. This could result from its isolated distribution in high-altitude regions, where primarily intraspecific hybridization has led to a slow domestication process [15].

### High heterozygosity observed in *P. ussuriensis*

The *P. ussuriensis* exhibits a high level of heterozygosity. We believe this may be primarily due to the fact that cultivated *P. ussuriensis* is mainly derived from hybridization between *P. pyrifolia*/P. bretschneideri and wild *P. ussuriensis* (rather than being predominantly obtained through the domestication of wild populations). There is significant genetic introgression between *P. pyrifolia*/*P. bretschneideri* and cultivated *P. ussuriensis* (Fig. S9A), and these introgressions could be some of the main reasons contributing to the higher heterozygosity in cultivated *P. ussuriensis*. Additionally, *P. ussuriensis* is primarily distributed in high-altitude regions, where it is subjected to stronger natural environmental selection pressures. Compared to other populations, it experiences less human-driven selection pressure, which may result in slower domestication and consequently higher heterozygosity and nucleotide diversity.

High heterozygosity often masks recessive deleterious mutations in the genome. Both classical genetics and modern molecular evolutionary studies have demonstrated that inbreeding/selfing depression and heterosis are primarily driven by the presence of recessive deleterious mutations within populations [59]. As selective breeding progresses, some recessive, masked deleterious mutations may be retained due to the hitchhiking effect, and may even manifest their deleterious effects, potentially introducing additional hidden genetic burdens to breeding programs [12]. This highlights the complexity of heterozygosity in shaping genetic fitness. Recent research has further revealed that selective sweeps at heterozygous loci in poplars play a dual role. On one hand, they modulate gene expression patterns, coordinately regulating growth, development, and environmental adaptability. On the other hand, they enhance adaptability by reducing the number of deleterious mutations in stress-response pathway genes [60]. These findings underscore the multifaceted impact of heterozygosity on species adaptability. In our study, we observed that *P. ussuriensis* exhibits notably high heterozygosity. However, the specific implications of this elevated heterozygosity on its adaptability remain to be further explored. This warrants deeper investigation to understand how heterozygosity influences the adaptive evolution and ecological fitness of *P. ussuriensis*.

### Multifaceted impacts of deleterious mutations

Deleterious mutations near target genes may be retained through selective breeding, as described by the domestication hypothesis [6, 58]. These mutations can negatively impact gene expression, protein structure, and agronomic traits. For example, in maize, deleterious SNPs near key genes affect growth and yield under certain conditions [61]. While most research on deleterious mutations focuses on annual crops [6–9, 12], our study contributes to the relatively limited understanding of these mutations in perennial fruit trees.

Despite their negative reputation, deleterious mutations are not universally detrimental to agronomic traits. For example, in walnuts, a nonsynonymous SNP in *JrFAD2* alters fatty acid composition, while another SNP in JrANR influences pellicle coloration [31]. Similarly, in pears, a 14-bp deletion in the *PbrBBX24* gene promotes anthocyanins biosynthesis and dwarfism, traits advantageous for fruit quality and cultivation [62]. Our findings on deleterious nonsynonymous mutations in *PyMYC2* gene lead to increased stone cell content. Notably, most *P. pyrifolia*/*P. bretschneideri* cultivars lack these three deleterious mutations, which aligns with the observed lower stone cell content in cultivated varieties compared to wild relatives. These results demonstrate that so-called ‘deleterious mutations’ are defined at the genetic level, yet their phenotypic effects can be multifaceted. While potentially detrimental to the plant itself, they may confer agronomic benefits for human cultivation. In conclusion, the impact of deleterious mutations is context dependent, with implications varying based on specific production needs. While these mutations may impair gene function, their functions on agronomic traits can sometimes be beneficial, providing opportunities for targeted breeding strategies.

## Materials and methods

### Plant materials and data sources

A total of 232 pear germplasm was collected, including 15 white pears, 47 cultivated Chinese sand pears, 15 wild Chinese sand pears, 10 cultivated Nashi pears, 22 wild Nashi pears, 5 Xinjiang pears, 28 other Asian wild pears, 55 cultivated European pears, and 35 wild European pears. Among them, resequencing data for 113 pear samples were sourced from our previous study [15], and resequencing data for 35 pear samples were obtained from our recent work [46]. Additionally, 84 samples were newly collected and sequenced for this study. We rapidly froze the peels of the remaining 84 samples in liquid nitrogen and stored them at −80°C for whole-genome sequencing.

### DNA extraction and sequencing

DNA was extracted from the pear samples using the CTAB method and a DNA isolation kit (Tiangen, Beijing, China). The extracted DNA (OD260/280 = 1.8–2.0; total content > 6 μg) was used to construct whole-genome sequencing libraries. Whole-genome sequencing (WGS) libraries were sequenced using the Illumina HiSeq 4000 system (150 bp paired-end reads).

### SNP calling

The quality of the WGS data was checked using the FastQC (v0.11.9) package with default parameters [63]. Trimmomatic (v0.39) [64] was used to trim low-quality reads and adapter sequences with the following parameters: ‘adapters.fa:3:30:10 SLIDINGWINDOW:4:20 MINLEN:50’. Clean data were mapped to the ‘Cuiguan’ genome using BWA software (v0.7.17-r1188) with parameters: ‘mem -t 15 -M -k 32’. The sam files were converted to bam files and sorted using SAMtools (v1.6) [65]. Subsequently, the ‘MarkDuplicates’ function of Picard software was used to filter reads mapped to multiple locations. SNPs were called using the HaplotypeCaller, CombineGVCF, and GenotypeGVCFs functions of GATK software (v4.4.0.0), and SNPs were identified using the VariantFiltration function with the following parameters: ‘QD < 2.0, FS > 60.0, MQ < 40.0, MQRankSum < −12.5, ReadPosRankSum < −8.0, -cluster 3 -window 10’. SNPs were then filtered using VCFtools (v0.1.16) based on a missing rate ≤ 70% and MAF ≥ 0.05, resulting in a total of 9 909 773 SNPs. All SNPs were annotated using the table_annovar.pl function in the ANNOVAR software [66].

### Population genetics analysis

First, we used the Reseqtools package to extract fourfold degenerate sites (4DTV) from the ‘Cuiguan’ genome [67]. Based on the maximum likelihood method, we constructed a phylogenetic tree using Fasttree software (v2.1.11) [68], and visualized and adjusted the tree using iTOL [69]. PCA analysis was performed using plink software (v1.90b6.21) and visualized with the R package ggplot2. Population structure was inferred using inferred using ADMIXTURE software (v1.3.0) with K values ranging from 2 to 10 [70].

### Linkage disequilibrium and diversity analysis

LD decay statistics for different populations were calculated using PopLDdecay software (https://github.com/BGI-shenzhen/PopLDdecay) with parameters: ‘-MaxDist 500 -MAF 0.05’, and LD statistics for all populations were visualized using the Plot_MultiPop.pl function. Nucleotide diversity (π) was calculated using VCFtools (v0.1.16) [71] with a sliding window of 10 kb and a step size of 5 kb, and population differentiation index (*F*st) was calculated in the same way to compare the degree of population differentiation between wild and cultivated germplasm.

### Genome-wide selective sweep analysis

To identify selective signals during the domestication process in different pear germplasm, we identified selective signals in cultivated populations with wild pear populations as the background. We conducted a cross-population composite likelihood ratio test based on a sliding window of 10 kb and a step size of 5 kb using XP-CLR software [72], with the top 5% of genome regions with the highest XP-CLR scores considered as selective regions.

### Amplification of target gene fragment

Single-stranded cDNA was synthesized from different pear varieties using the RNApure Fast Plant Kit (ComWin Biotech, Jiangsu) to extract RNA, and the TransScript® One-Step gDNA Removal and cDNA Synthesis SuperMix (TransGen Biotech, Beijing) for reverse transcription. PCR amplification was performed using cDNA from different pear varieties as the template. The PCR reaction system (50 μl) included: 10 μl of 5× Buffer, 4 μl of dNTPs, 1 μl of each primer, 1 μl of cDNA, 2 μl of GXL enzyme, and 6 μl of ddH2O. The reaction procedure was predenaturation at 95°C for 5 min; 34 cycles of denaturation at 95°C for 30 s, annealing at 58°C for 30 s, and extension at 72°C for 60 s; final extension at 72°C for 10 min. Subsequently, the target bands were detected by electrophoresis.

### GO and KEGG annotations

We used the TBtools software [73] to perform KEGG and GO enrichment analyses on genes from different pear germplasm selected during their respective domestication intervals. The GO ontology data were sourced from the ‘go-basic.obo’ file, accessible at http://purl.obolibrary.org/obo/go/go-basic.obo. Similarly, the KEGG backend file titled ‘TBtoolsKEGGMap.DB’ was obtained from https://tbtools.cowtransfer.com/s/566e88227a0045. The background files for both GO and KEGG associated with the ‘Cuiguan’ genome were acquired using EGGNOG-Mapper, available online at http://eggnog-mapper.embl.de/. The analysis of the GO and KEGG data was performed using the ‘ggplot2’ package in the R programming environment.

### Identification and analysis of deleterious mutation sites

In this study, we primarily predicted whether amino acid substitutions affect protein function based on sequence homology and the physical properties of amino acids, thereby determining whether a mutation at a specific genomic site is deleterious. Since the ‘Cuiguan’ pear is not currently included in the SIFT database, we first manually constructed its database using the make-SIFT-db-all.pl function of the SIFT 4G software, and then annotated all SNP sites using the SIFT4G_Annotator.jar function. Sites with a SIFT score below 0.05 were considered deleterious to the genome [74].

To verify the overall accuracy of the SIFT 4G predictions, we also estimated the deleterious mutation burden at each site in the pear genome based on Genomic Evolutionary Rate Profiling (GERP) scores [75]. GERP scores reflect the intensity of purifying selection based on whole-genome alignments across multiple plant species. We obtained soft repeat-masked genomes for the following species from the RefSeq database: *A. thaliana*, *Glycine max*, *Nicotiana tabacum*, *O. sativa*, *Populus trichocarpa*, *Prunus persica*, *Rosa chinensis*, *Vitis vinifera*, and *Zea mays*. Additionally, we performed de novo repeat prediction for the ‘Cuiguan’ genome using RepeatModeler [76] and masked the repetitive sequences using RepeatMasker [77], obtaining the ‘Cuiguan’ soft repeat-masked genome.

Using the LASTz/MULTIz method (http://genomewiki.ucsc.edu/index.php/DoBlastzChainNet.pl) and the python script pairwise_genome_alignment.py (https://github.com/wen-chen/naughty_monkey/tree/main/pairwise_genome_alignment), we performed multi-genome alignments of the 10 soft repeat-masked genomes. The phylogenetic tree was obtained from the Dryad database, and the topology of the 10 species was extracted using the ete3 toolkit (v3.1.1) [39]. We ran phyloFit to estimate the nonconserved model using 4d sites of chromosome 15 in the ‘Cuiguan’ genome. Finally, we calculated GERP scores for the pear genome using the gerpcol function of the GERP++ Programs, considering sites with a GERP score greater than 2 as deleterious SNPs.

The homozygous state of each SNP site was obtained from the annotation results of ANNOVAR. The calculation method for the frequency of deleterious SNPs in the domestication interval is: (number of deleterious SNPs in the domestication interval of the germplasm)/(total number of deleterious SNPs in the whole genome of the germplasm) × 100%. The heterozygosity (F) of each pear individual was calculated using Plink (v1.90b6.21) [78], with the formula: 


\begin{equation*}F = (O(hom)-E(hom)) / (N-E(hom))\end{equation*}

where *O*(hom) is the observed number of homozygous SNPs, *E*(hom) is the expected number of homozygous SNPs, and *N* is the total number of genotyped sites in the individual.

### Gene introgression analysis

To explore the reasons for changes in deleterious SNPs in the *P. ussuriensis* population, we conducted a gene introgression analysis specifically for the *P. ussuriensis* population. The introgression of gene fragments from cultivated Asian pears to cultivated *P. ussuriensis* was identified using the four-taxa fd statistical method.

We used wild European pears as the outgroup, with wild *P. ussuriensis* and cultivated *P. ussuriensis* as the gene recipient populations P1 and P2, respectively, and cultivated Asian pears as the gene donor population P3. In the absence of gene introgression, the ABBA and BABA allele configurations in (((P1, P2), P3), O) should be the same; however, when there is gene introgression between P3 and P2, ABBA will increase relative to BABA. We used the freq.py program to calculate the frequency of derived alleles for each polymorphic site in the genome of each population, and the jackknife.R program to calculate the average D statistic and the Z score of the D statistic. When the D statistic is greater than 0, we consider there to be gene introgression between P3 and P2. When the D *Z* score is greater than 4, we consider the introgression to be significant. We then used the ABBABABAwindows.py program to calculate the genome-wide Fd statistic, using a 10 kb sliding window with a 5 kb step. Windows with fewer than 100 biallelic SNPs, windows with negative D statistics, and windows with Fd values greater than 1 were ignored. The Fd statistics for the whole genome were visualized using *R*, with the top 5% Fd scores considered as gene introgression regions from cultivated Asian pears to *P. ussuriensis*.

### Genome-wide association analyses

Based on the previous data and methodology [45], a GWAS was conducted using EMMAX. The cutoff was used as *P* = 1e−5.

### Genetic transformation of pear callus

The transformation of pear callus was conducted as previously described [62]. Pear callus was infected with Agrobacterium cells (OD_600_ = 0.6) carrying the 35S::PyMYC2-GFP, followed by incubation in liquid MS medium for 20 min. After filtration, the callus was cultured on co-cultivation medium for 2 days and then transferred to MS medium containing 20 mg L^−1^ hygromycin, incubated in the dark at 24°C. Callus regeneration was subcultured every 2–3 weeks. After three successive subcultures, the calli were transferred to induction MS medium supplemented with 10 μmol·L^−1^ EBR and incubated for 3 weeks [79]. Subsequently, the calli were subjected to staining using Wiesner’s reagent.

### RNA extraction and RT-qPCR

Total RNA was isolated from pear fruit tissues using a polysaccharide-polyphenol optimized extraction kit (FUJI, China). Genomic DNA contamination was eliminated using the One-Step gDNA Removal Kit (Vazyme, China), followed by first-strand cDNA synthesis with 1 μg RNA template (Vazyme cDNA Synthesis Kit). Quantitative PCR was performed on a LightCycler 480 system (Roche, USA) with SYBR Green Master Mix, using primers listed in Supplementary Table S1. The 2 − ΔΔCt method [80] was applied. Each experiment included at least three biological replicates.

### Lignin analysis

The overexpressed pear callus and the control pear callus were dried, ground into powder using liquid nitrogen, and then the lignin content was measured according to previously reported methods [81].

## Supplementary Material

Web_Material_uhaf140

## Data Availability

Data supporting the findings of this work are available within the paper and its Supplementary Information files. Raw genome re-sequencing reads have been deposited into the NCBI sequence read archive(SRA) (https://www.ncbi.nlm.nih.gov/sra/) under BioProject accession number of PRJNA1258372.
