# Supplementary material for: Genomic insights into deleterious mutations and their impact on agronomic traits during pear domestication
Source: Hortic Res. 2025 May 29;12(9):uhaf140. doi: 10.1093/hr/uhaf140 (PMC12313341; doi:10.1093/hr/uhaf140)
Supplement: Web_Material_uhaf140 [file web_material_uhaf140.zip › Supplementary Figure.docx]

**Additional file 1**


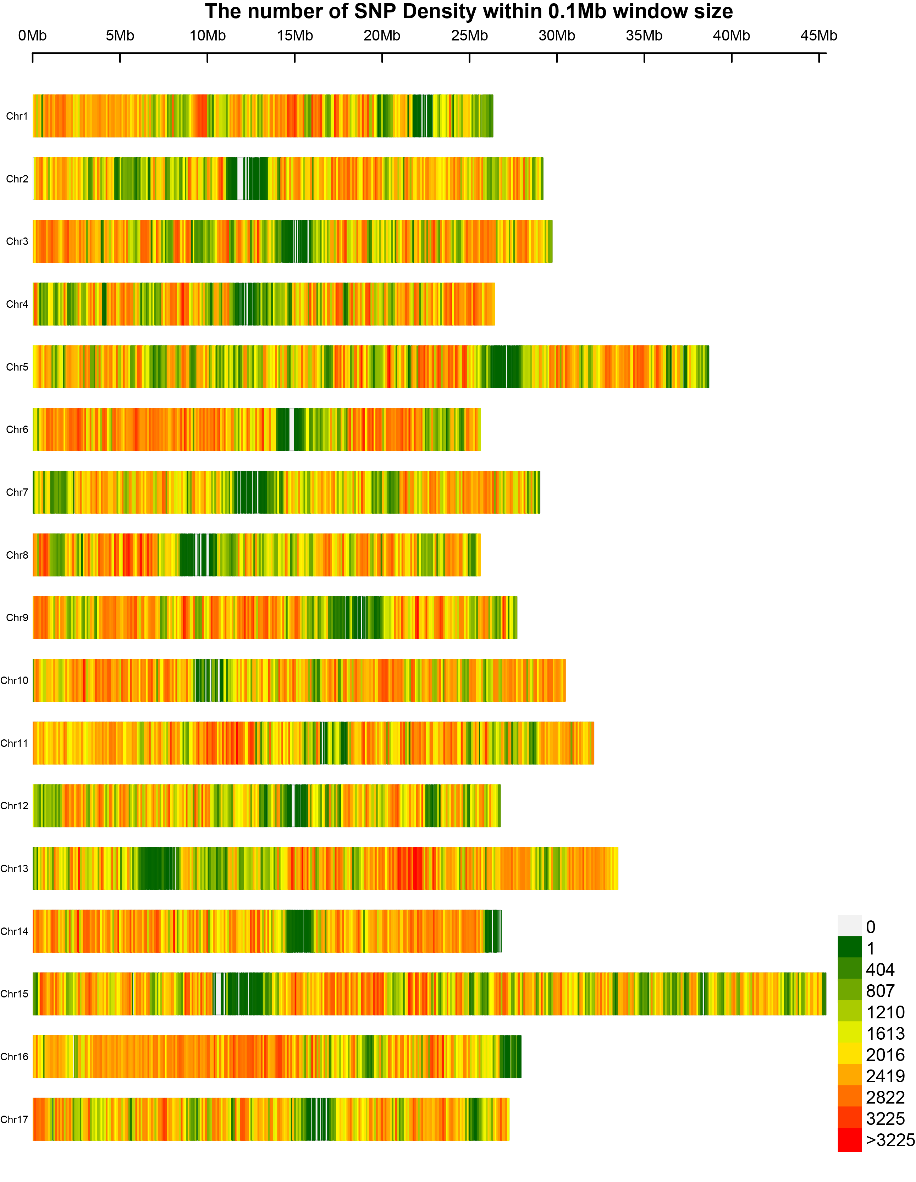


**Figure S1:** Heat map showing the SNP density distribution across the genome with a window size of 100 kb. Colors closer to red indicate higher SNP density, while colors closer to green represent lower SNP density within a window.


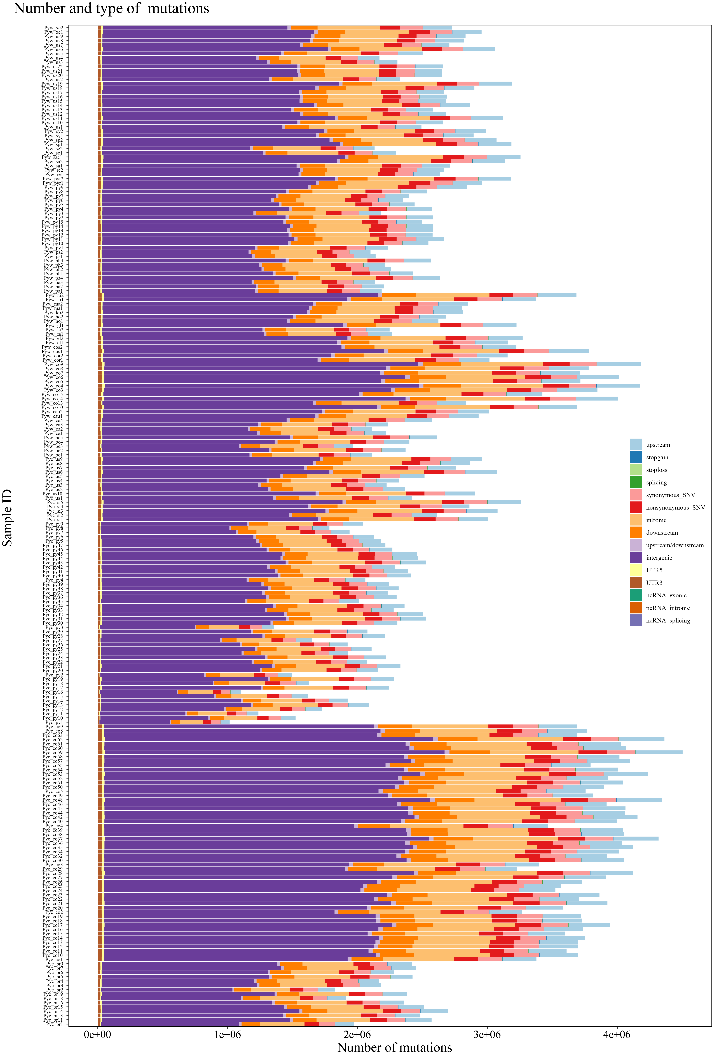


**Figure S2:** Histograms displaying the whole-genome SNP compositions across different samples. Upstream: Variants overlapping a 1-kb region upstream of the transcription start site. Stopgain: Variants introducing a stop codon. Stoploss: Variants changing a stop codon to a non-stop codon. Splicing: Variants within 2 bp of a splicing junction. Synonymous_SNV: Variants without amino acid changes. Nonsynonymous_SNV: Variants causing amino acid changes. Intronic: Variant located within introns. Downstream: Variant overlapping a 1-kb region downstream of the transcription start site. Upstream/downstream: Variant overlapping both upstream and downstream 1-kb regions. Intergenic: Variants located between genes. UTR5 and UTR3: Variants overlapping the 5′ and 3′ untranslated regions, respectively. ncRNA_exonic: Variants in the non-coding RNA exons. ncRNA_intronic: Variants in the non-coding RNA introns. ncRNA_splicing: Variant within 2 bp of a non-coding splicing junction.


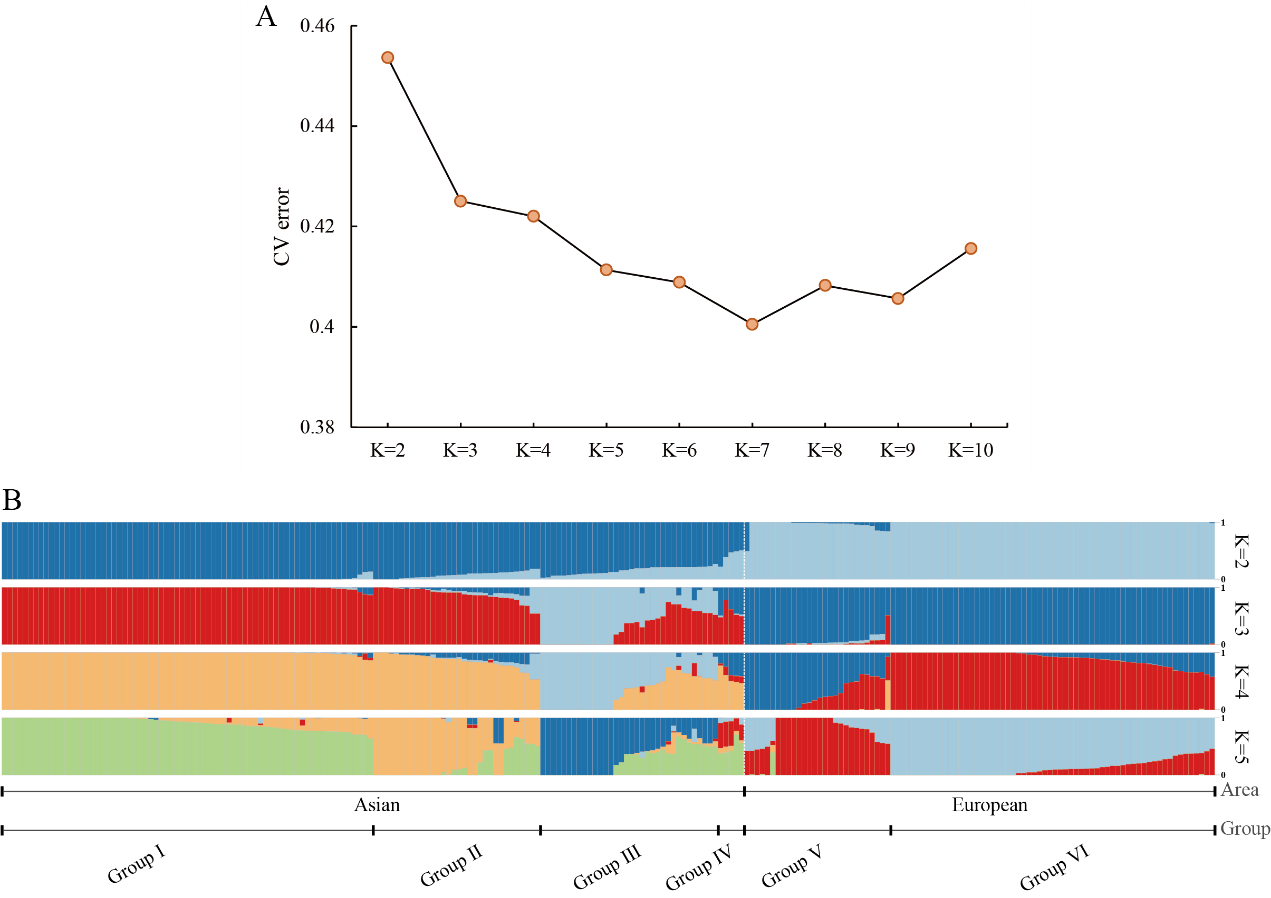


**Figure S3:** **(A)** Cross-validation error value for different K values in STRUCTURE analysis. K = 7 appears optimal based on error values; however, clustering results are more consistent with K = 5. **(B)** Population structure analysis of 232 pear germplasm accessions with K = 2 to 5). Each color represents a distinct population, with individual accessions displayed as vertical lines.


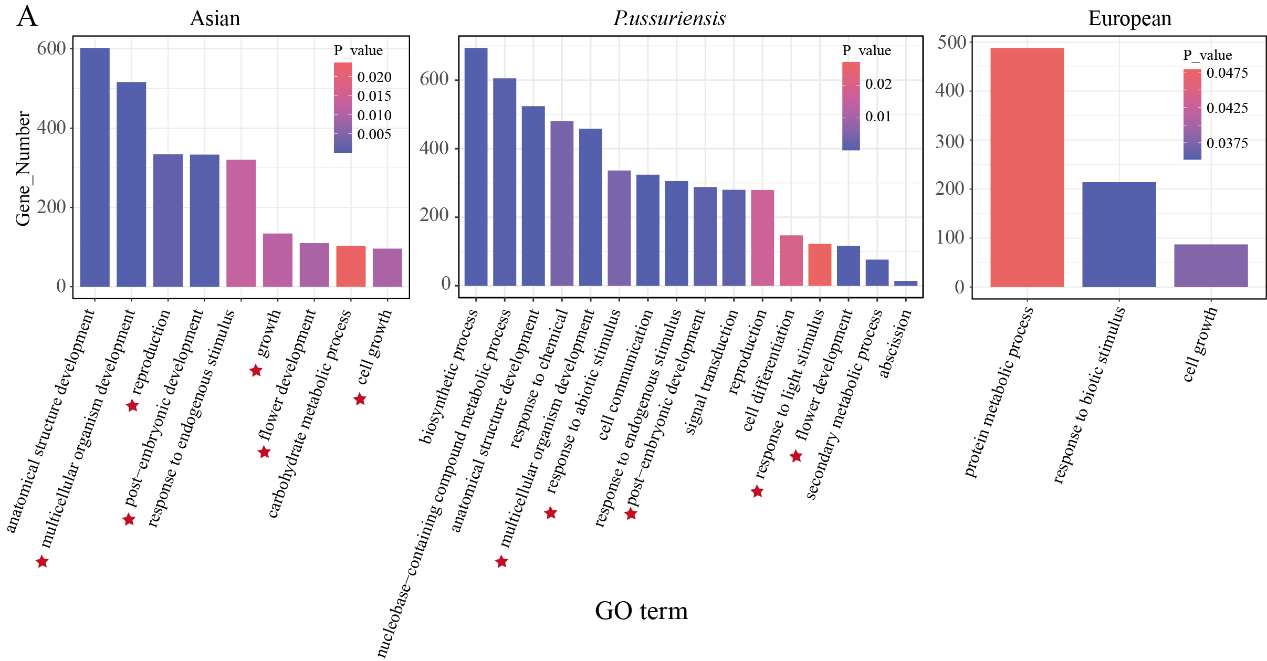


**Figure S4:** GO enrichment analysis of genes in selective sweep regions. **(A)** Bar charts illustrating GO enrichment analysis, from left to right, are Comparison A (*P. pyrifolia*/*P. bretschneideri* VS wild Asian), Comparison B (cultivated *P. ussuriensis* VS wild *P. ussuriensis*), and Comparison C (cultivated European VS wild European). The x-axis shows GO terms, while the y-axis represents the number of genes. Bar color represents the p-value, with red indicating higher and blue indicating lower values.


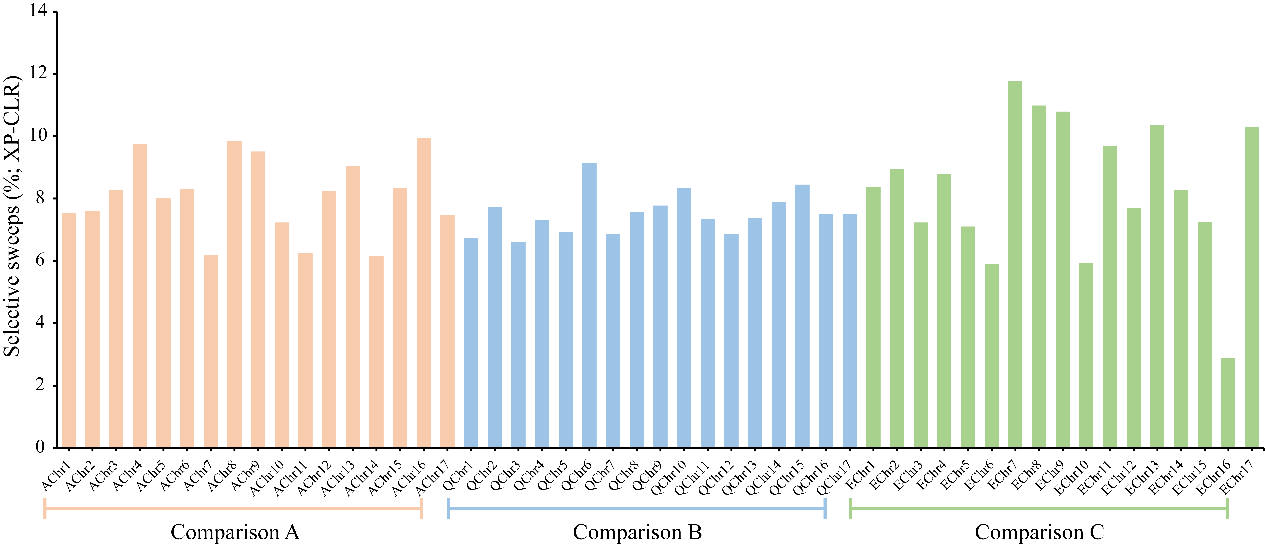


**Figure S5:** Proportion of selective sweep regions across 17 chromosomes for different populations. Orange indicates Comparison A (*P. pyrifolia*/*P. bretschneideri* vs. wild Asian), blue indicates Comparison B (cultivated *P. ussuriensis* vs. wild *P. ussuriensis*), and green represents Comparison C (cultivated European vs. wild European). Chromosomes 1 - 17 from left to right, with the y-axis representing the proportion of each chromosome’s selective sweep region relative to its total length.


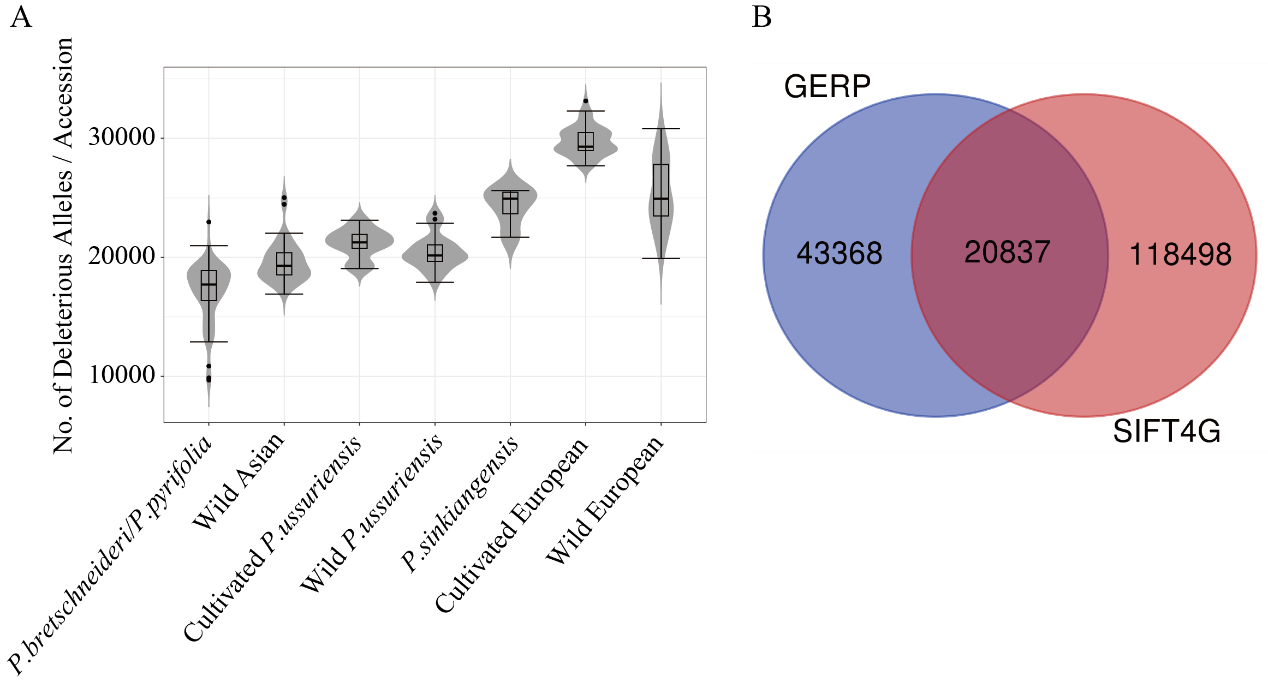


**Figure S6:** **(A)** Number of deleterious mutation sites in various pear populations identified using GERP software. The x-axis represents populations, and the y-axis shows the number of deleterious mutations, consistent with trends observed using SIFT4G software. **(B)** Venn diagram comparing the number of deleterious variants identified by GERP and SIFT4G. From left to right are the number of deleterious variants specific to GERP, the number of deleterious variants shared by GERP and SIFT4G, and the number of deleterious variants specific to SIFT4G.


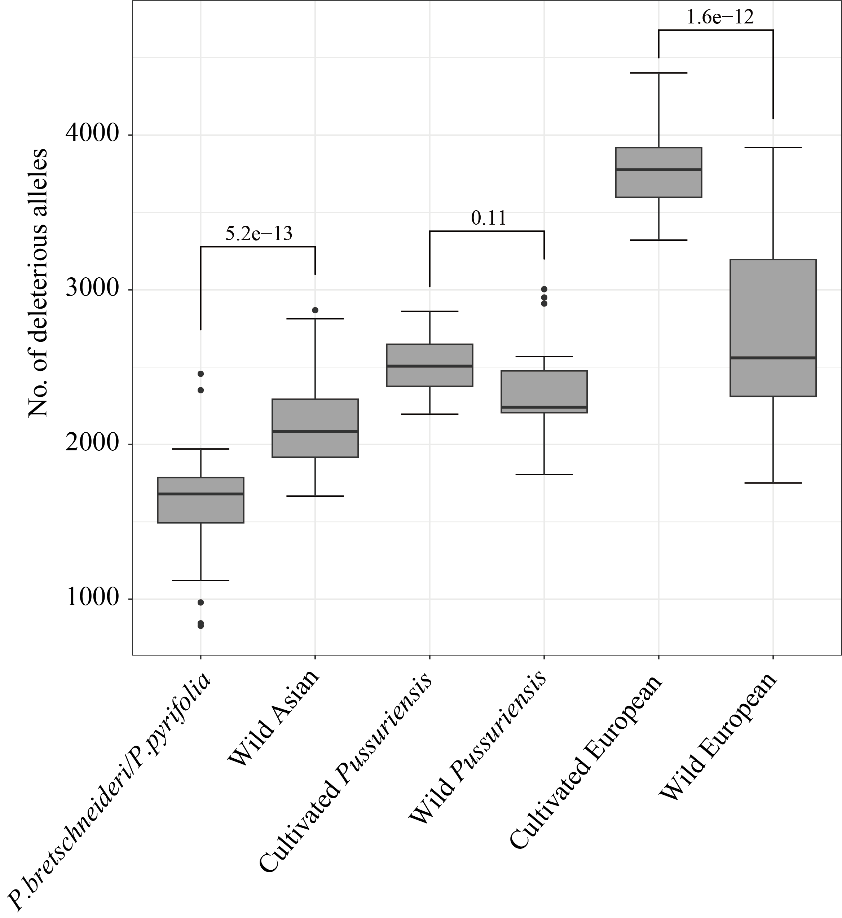


**Figure S7:** Number of deleterious mutations in selective sweep regions across pear populations. The x-axis shows population groups, while the y-axis displays deleterious mutation counts. Results align with whole genome observations, with significant differences in Comparison A (*P. pyrifolia*/*P. bretschneideri* VS wild Asian) and Comparison C (cultivated European VS wild European), significant differences in Comparison B (cultivated *P. ussuriensis* VS wild *P. ussuriensis*).


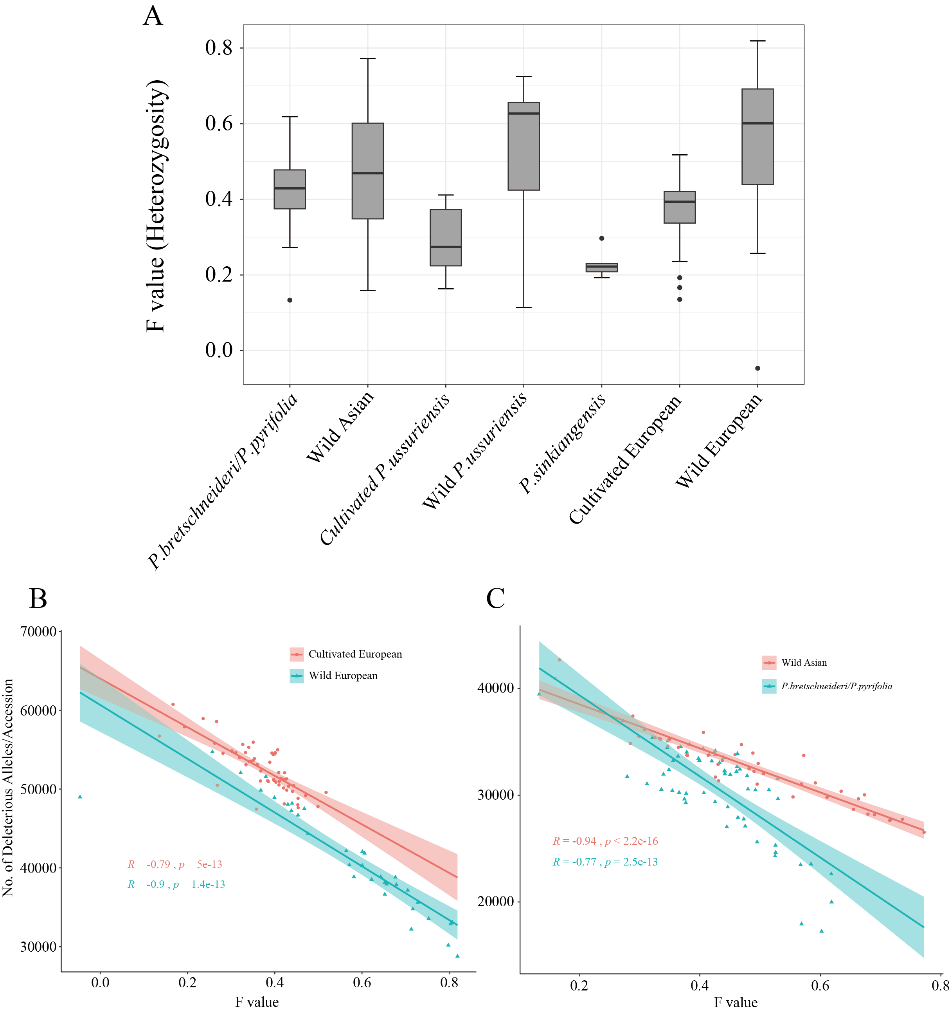


**Figure S8:** Heterozygosity statistics across pear populations. **(A)** The x-axis shows populations, and the y-axis represents heterozygosity (F value). Smaller F values indicate higher heterozygosity. **(B)** Linear relationships between heterozygosity (F value) and deleterious mutation numbers in Comparison C (cultivated European VS wild European) and Comparison A (*P. pyrifolia* / *P. bretschneideri* VS wild Asian), respectively. Blue represents *P. pyrifolia / P. bretschneideri,* and red represents wild Asian pears.


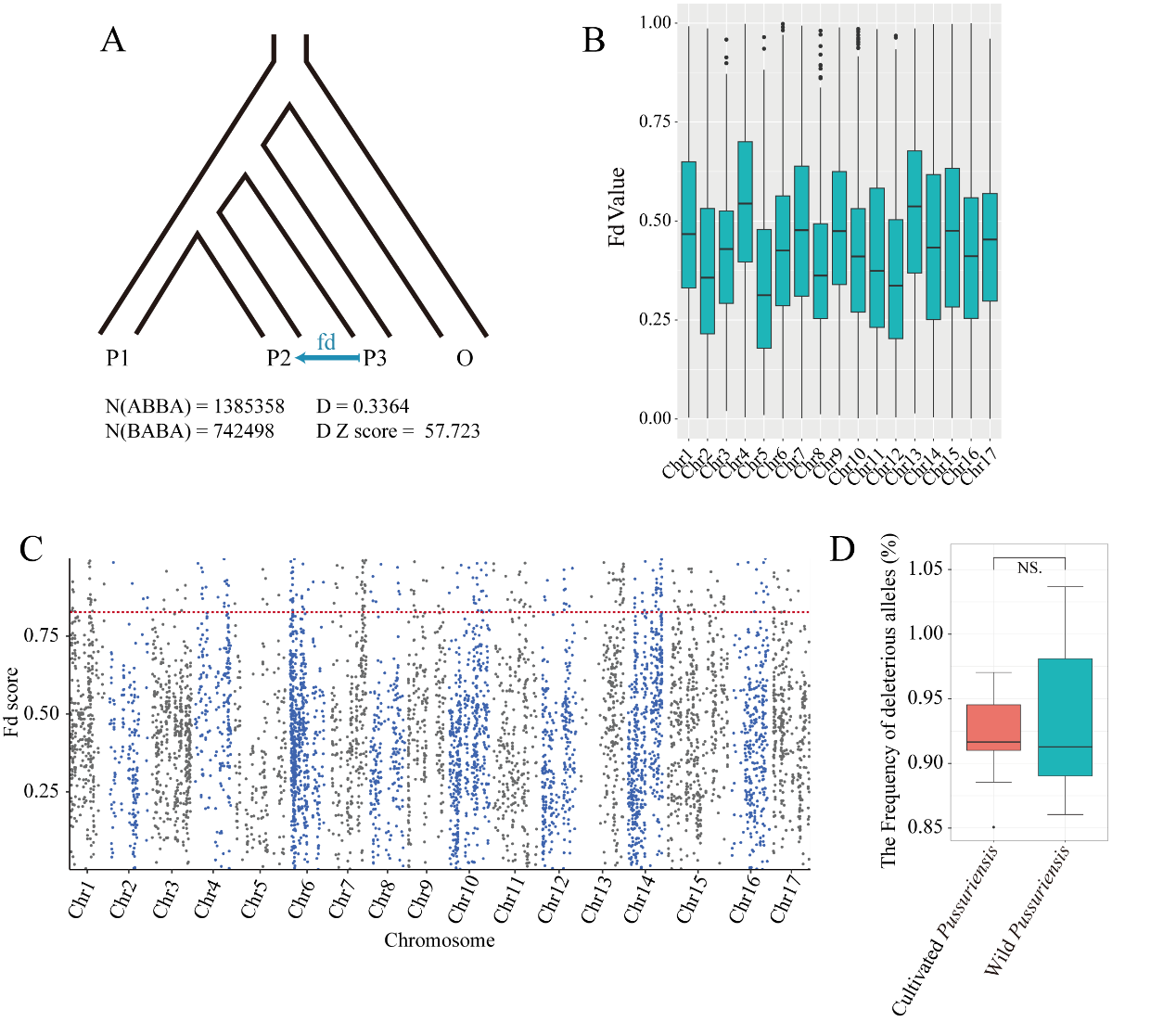


**Figure S9:** Introgression analysis in *P. ussuriensis*. **(A)** ABBA-ABAB test results, with P1 and P2 as closely related populations (wild and cultivated *P. ussuriensis*, respectively), P3 as the source population (*P. pyrifolia / P. bretschneideri)*, and O as the outgroup (wild European pears). D > 0 indicates introgression between P2 and P3. **(B)** Box plot showing Fd statistics for chromosomes. Higher Fd values indicate stronger introgression. **(C)** Sliding window analysis of introgression intervals (10 kb). Intervals with Fd > 0.826920 are considered introgression intervals. **(D)** Frequency of deleterious mutations within introgression intervals of wild and cultivated *P. ussuriensis*.


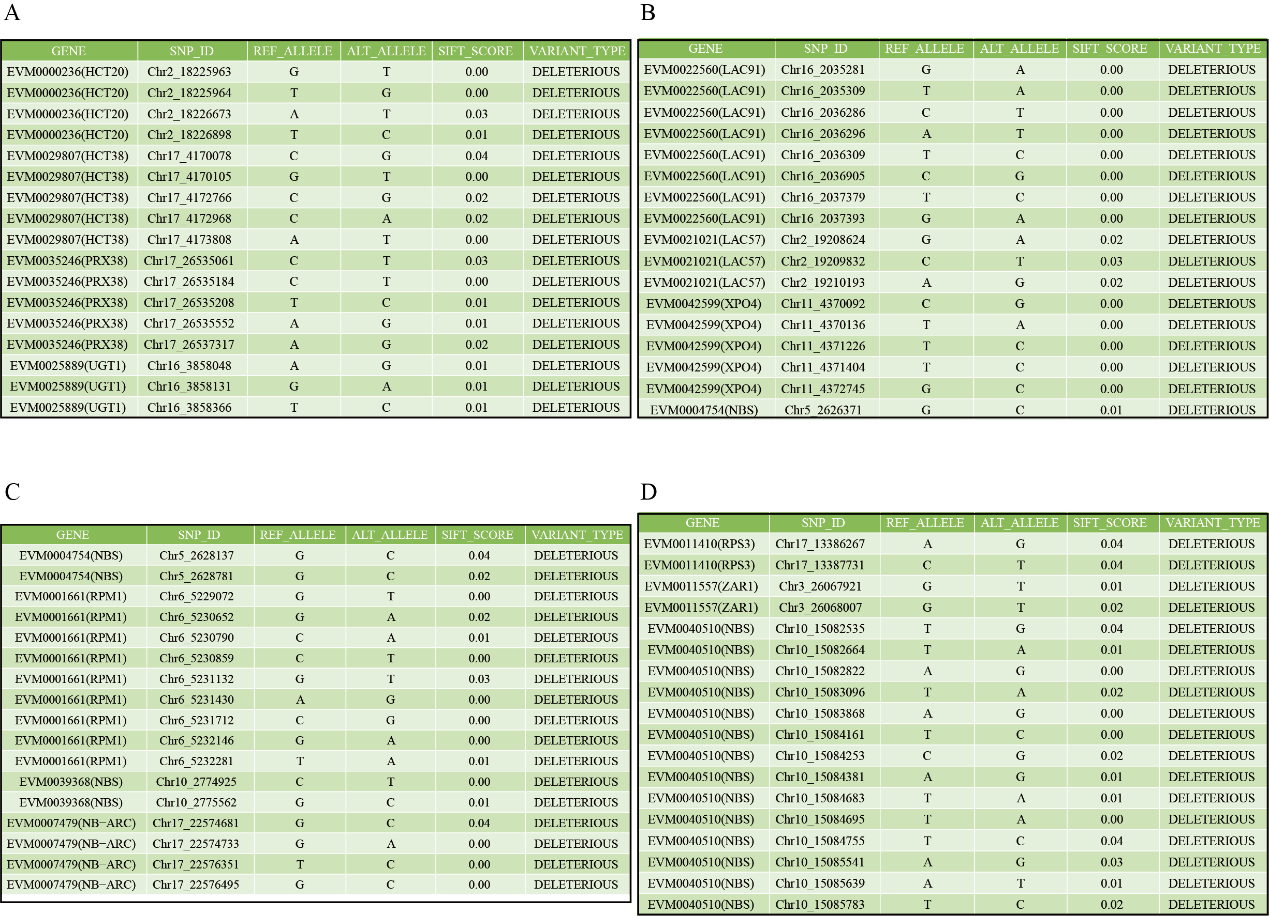


**Figure S10:** Deleterious mutations in specific DHGs validated by multi-omics datasets. **(A-D)** Panels display deleterious mutation sites across 14 genes, including base changes, SIFT scores, and variant types.


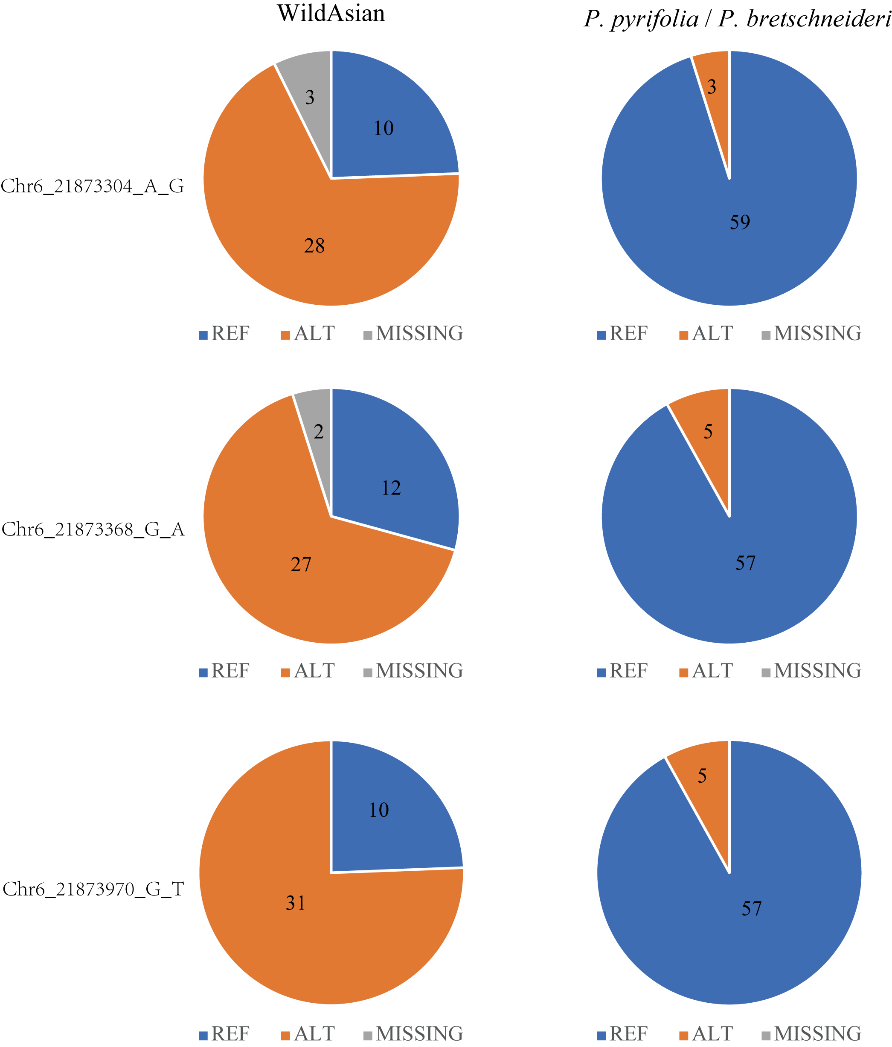


**Figure S11:** Genotype proportions of three conserved SNP in *PyMYC2* among 232 pear samples. From top to bottom, SNP1 (Chr6_21873304), SNP2 (Chr6_21873368), and SNP3 (Chr6_21873970) are shown. Blue represents REF genotypes (non-deleterious), orange represents ALT genotypes (deleterious), and gray indicates absence.


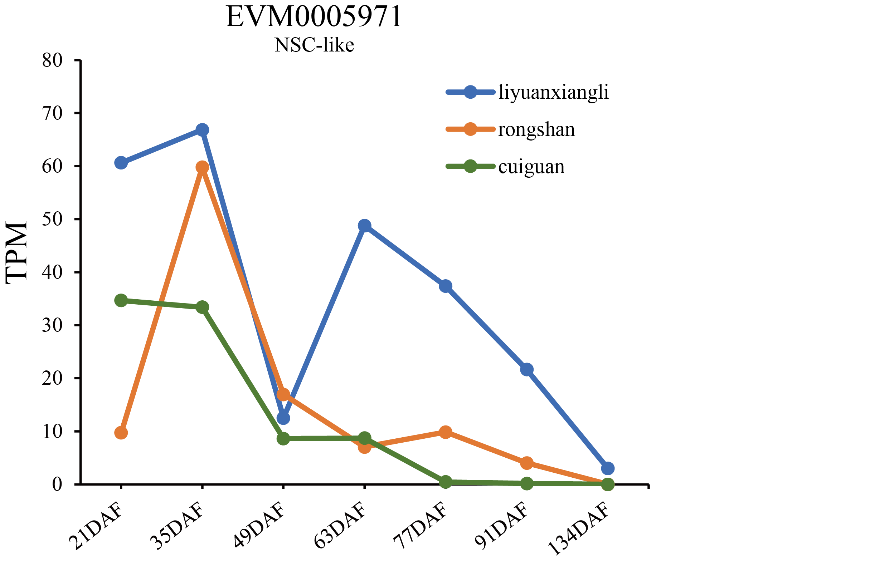


**Figure S12:** Expression levels of EVM0005971 (*PbrNSC*-like) during fruit development. The x-axis represents days after flowering (21 to 134 days), and TPM values on the y-axis represent expression trends for high (blue), medium (orange), and low (green) stone cell content varieties: ‘Liyuanxiangli,’ ‘Rongshan,’ and ‘Cuiguan,’ respectively.


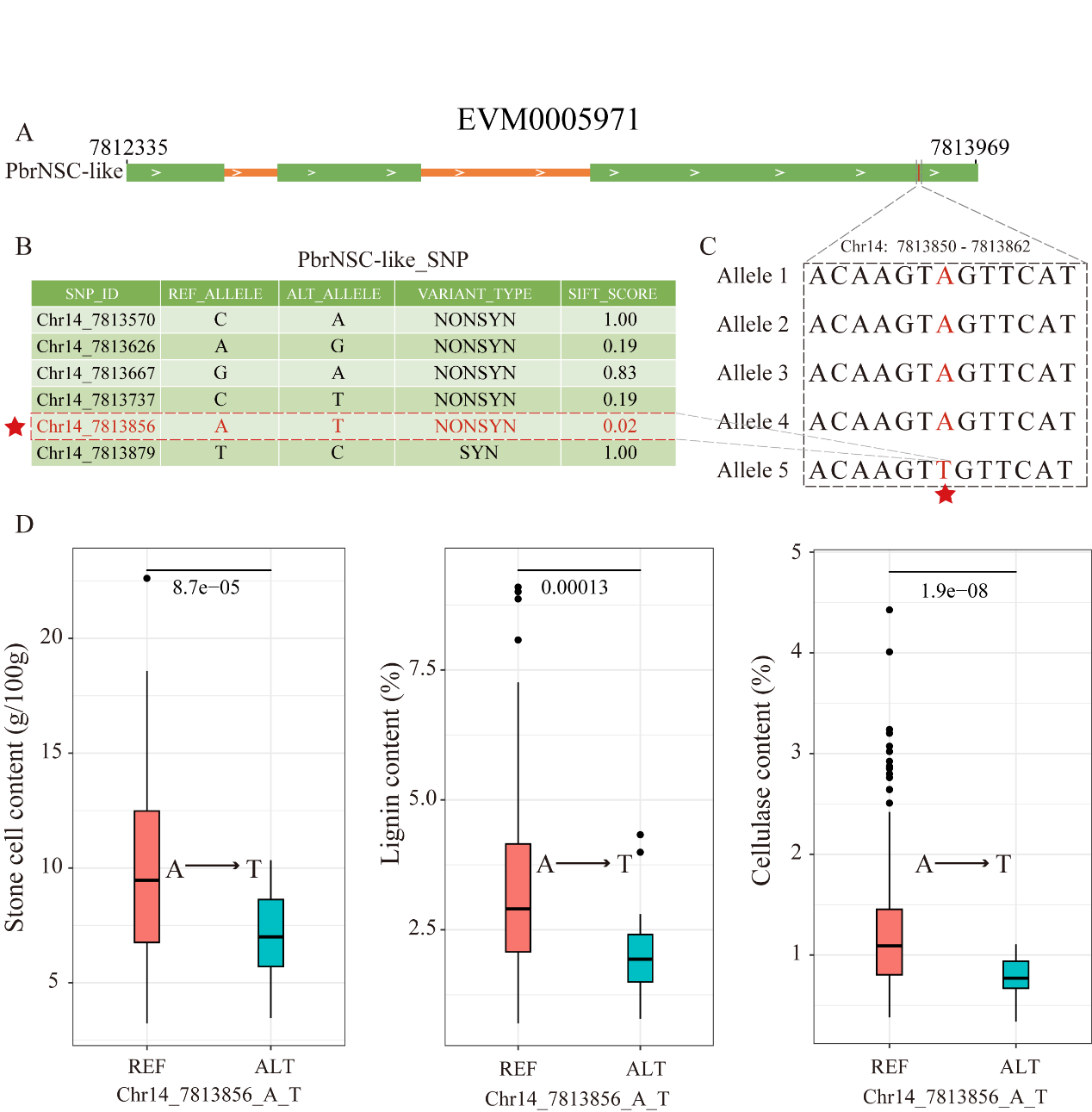


**Figure S13:** Deleterious mutation in *PbrNSC-like* (EVM0005971.1). **(A)** Gene structure schematic showing deleterious mutation sites. The yellow segments represent genomic regions, the green segments indicate CDS regions and the red line marks the deleterious mutation sites. **(B)** Information on SNPs in *PbrNSC-like* and SIFT scores, with deleterious mutation sites highlighted in red. **(C)** Genotypes at Chr14:7813850-7813862, where the red area indicates a potential deleterious mutation (A to T). **(D)** From left to right, statistics of stone cells, lignin, and cellulose content based on genotypes REF (without deleterious mutation) vs. ALT (with deleterious mutation) at Chr14_7813856.
